# Surface premelting and melting of colloidal glasses

**DOI:** 10.1126/sciadv.adf1101

**Published:** 2023-03-17

**Authors:** Qi Zhang, Wei Li, Kaiyao Qiao, Yilong Han

**Affiliations:** ^1^Department of Physics, Hong Kong University of Science and Technology, Hong Kong, China.; ^2^Hong Kong University of Science and Technology, Shenzhen Research Institute, Shenzhen 518057, China.

## Abstract

The nature of liquid-to-glass transition is a major puzzle in science. A similar challenge exists in glass-to-liquid transition, i.e., glass melting, especially for the poorly investigated surface effects. Here, we assemble colloidal glasses by vapor deposition and melt them by tuning particle attractions. The structural and dynamic parameters saturate at different depths, which define a surface liquid layer and an intermediate glassy layer. The power-law growth of both layers and melting front behaviors at different heating rates are similar to crystal premelting and melting, suggesting that premelting and melting can be generalized to amorphous solids. The measured single-particle kinetics reveal various features and confirm theoretical predictions for glass surface layer.

## Teaser

The in situ observation with single-particle kinetics reveals two surface layers and premelting behaviors on glass surface.

## INTRODUCTION

Glass melting is not a reverse process of liquid-to-glass transition, i.e., vitrification or glass transition. For example, strains can exist in the solid parent phase during melting but not in the liquid parent phase during vitrification (*1*); the heat capacity of glass exhibits hysteresis upon cooling and heating (*2*, *3*); rapidly quenching liquid produces glass, whereas heating glass at the same rate may produce crystal, i.e., devitrification, rather than melting (*4*). In contrast to the intensively studied glass transition, the study about glass melting is at the preliminary stage (*5*–*11*). For ordinary glasses, liquids usually homogenously form within bulk via a nucleation-like process and grow with Avrami-type kinetics (*7*, *9*), whereas melting at free surfaces (i.e., vapor-solid interfaces) is negligible compared with melting in large bulk (*5*). By contrast, ultrastable glasses exhibit heterogeneous surface melting (*5*, *8*, *10*, *12*–*14*). A crystal surface usually forms a thin liquid layer slightly below the melting temperature *T*_m_, i.e., surface premelting (*15*). Such surface liquid propagates into bulk when *T* ≥ *T*_m_, i.e., surface melting, and preempts melting from within. Surface melting has been studied in atomic and molecular ultrastable glasses (*5*, *8*, *10*, *16*) but lacks microscopic measurement, comparison with crystals, and a quantitative theory. In glass melting, the glass transition temperature *T*_g_ plays the role of *T*_m_ in crystal melting (*2*, *5*, *8*, *17*), but whether glass exhibits surface premelting has not been explored.

Besides melting, glass surface behaviors, such as the surface mobile layer, are key topics in studies on molecular (*5*), metallic (*18*), and polymer (*19*) thin-film glasses. The surface mobile layer has been studied by comparing the behaviors of thin-film glasses with different thicknesses or other methods without single-particle resolution (*20*). Consequently, how different it is from a normal liquid and how local properties vary with depth remain unclear (*21*, *22*).

Colloids are outstanding model systems, because micrometer-sized particles and their thermal motions can be visualized and tracked by optical microscopy (*23*). Colloids have provided important microscopic information on bulk glasses (*24*), such as shear-induced bulk glass melting (*6*). The studies about glass surfaces are mainly near a fixed wall (*25*, *26*), and the free surface of glass has only been experimentally explored using repulsive colloidal particles during vapor deposition under gravity at a fixed temperature (*27*). Thermally induced bulk or surface melting has not been explored at the single-particle level, as it requires colloids with tunable attractions. Colloids with tunable attractions have been achieved by thermal-sensitive depletant (*28*), Casimir effect (*29*), DNA (*30*), and electric field (*31*). Here, we use attractive colloids whose dye-induced long-range attraction is tunable in 0 to 1 Boltzmann constant (*k*_B_*T*) (*23*) and measure the microscopic kinetics at different effective temperatures. Premelting and melting driven by weakening particles’ attractions are compared under slow and fast temperature changes for monolayer and multilayer samples.

## RESULTS

**Fig. 1.**
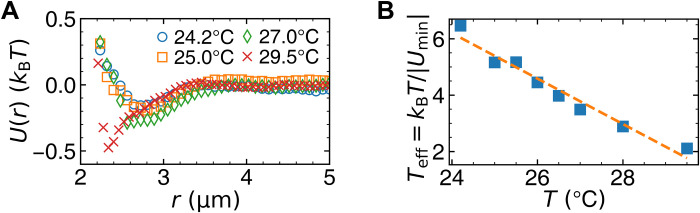
Pair potentials of PMMA spheres. (**A**) Pair potential *U*(*r*) as function of center-to-center distance at 27°C extracted from radial distribution function *g*(*r*) (fig. S1). (**B**) The effective temperature of 2.08-μm-diameter poly(methyl methacrylate) (PMMA) spheres *T*_eff_ = *k*_B_*T*/∣*U*_min_∣ decreases with the sample temperature *T*. *U*_min_ is the minimum of the measured *U*(*r*). Dashed line, linear fit; *k*_B_*T*, Boltzmann constant.

**Fig. 2.**
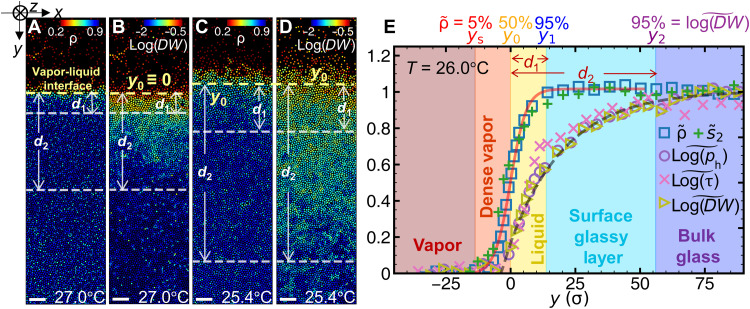
Surface premelting under slow temperature change. At 27.0° (**A** and **B**) and 25.4°C (**C** and **D**), particles in the monolayer are colored by ρ and log(*DW*), respectively. Scale bars, 20 μm. (**E**) At 26.0°C, the profiles of the structural parameters {$\tilde{\rho }(y)$ and ${\tilde{s}}_{2}(y)$} fitted by Eq. 3 (red solid curve); the profiles of the dynamic parameters {$\tilde{\log[\tau (y)]}$, $\tilde{\log[p_{h}(y)]}$, and $\tilde{\log[DW(y)]}$} fitted by Eq. 4 (black dashed curve). The five regimes (vapor, dense vapor, liquid, glassy layer, and bulk glass) have four interfaces labeled on the top *x* axis; their positions *y*_*s*,0,1,2_ are defined at $\tilde{\rho }=5,50,\mathrm{and}\;95\%$ and $\tilde{\log(DW)}=95\%$, respectively. *y* = 0 is defined as *y*_0_ at 27.0°C [yellow dashed lines in (A) and (B)].

We use 50:50 mixture of poly(methyl methacrylate) (PMMA) spheres with diameters of σ*_a_* = 2.08 μm and σ*_b_* = 2.74 μm to avoid crystallization. A dye is added to induce the depletion-like attraction between PMMA spheres (Fig. 1) (*23*). As temperature decreases, the dye is pumped to the unheated region due to thermophoresis, thus the attraction strength ∣*U*_min_∣ decreases (*23*), and the effective temperature *T*_eff_ = *k*_B_*T*/∣*U*_min_∣ increases linearly (Fig. 1B). Monolayer and multilayer colloids are confined between two centimeter-sized parallel glass plates with different separations in the *z* direction. Here, monolayer and multilayer refer to the layers in the *z* direction, whereas liquid or glassy layer refers to the layer in the *y* direction defined in Fig. 2A (fig. S3). The colloidal glasses are assembled via vapor deposition at a typical growth rate of 0.5 σ/*t*_0_, which well lies in the range of <100 for the formation of ultrastable molecular glasses (section S1 and movie S1) (*32*). *t*_0_ is the mean time for a particle on the vapor-liquid interface moving one mean diameter σ = (σ*_a_* + σ*_b_*)/2. Approximately 10^4^ particles are observed in the field of view by optical microscopy, and their Brownian motions are tracked by image analysis (*33*). The measurements below are made after the vapor deposition process. All the three monolayer samples show similar results. Experimental details are provided in Materials and Methods.

### Structural and dynamic parameters

Each particle *i* at depth *y* is characterized by two structural parameters [local density ρ*_i_* (Fig. 2, A and C) and two-body local excess entropy *s*_2*i*_ (*34*)] and three dynamic parameters [structural relaxation time τ(*y*), Debye-Waller factor *DW_i_* (Fig. 2, B and D) (*27*), and hop indicator function *p*_h*i*_ (*35*)] (Fig. 2E). $\rho_{i}\equiv \pi \sigma_{i}^{2}/(4A_{i})$ where *A_i_* is the area of particle *i*’s Voronoi polygon. *s*_2*i*_ reflects the number of inherent structures (*36*). The entropy of a system can be viewed as the sum of the ideal gas entropy and the excess entropy *s*_excess _. *s*_excess_ = *s*_2_ + *s*_3_ + *s*_4_ + ⋯ where *s_n_* is the *n*-body contribution to entropy (*37*). *s*_2_ contributes 80 to 90% of *s*_excess_ (*38*). Structural relaxation time τ, i.e., the decay time of self-intermediate scattering function *F_q_*, is measured from the fitting *F_q_*(*t*, *y*) ∼ *e*^−(*t*/τ)^β^^ (fig. S9). It is proportional to viscosity, which is a dynamic quantity. A particle’s vibration amplitude is described by

$${DW}_{i}(t)=\sqrt{\left\langle {\left[{\vec{r}}_{i}(t)-\langle {\vec{r}}_{i}(t)\rangle \right]}^{2}\right\rangle }/\sigma$$ (1)

where $\left\langle {\vec{r}}_{i}(t)\right\rangle$ is the average position during [*t* − *t*_0_/2, *t* + *t*_0_/2]. *t*_0_ is chosen as 30 s, because the out-of-cage time near the surface is about 30 s (fig. S10). Using other time intervals such as 100 or 200 s does not change the results (fig. S11). We also calculate the cage-relative Debye-Waller factor *DW*_cr_ in Eq. 11 based on the local coordinate of each particle’s neighboring partcicles (*39*). *DW* and *DW*_cr_ give similar results (fig. S23), indicating that the long-wavelength fluctuations (*40*, *41*) are not important to our results. Particle’s jumping ability can be described by the hop indicator function $p_{hi}(t)=\sqrt{{\left\langle {\left[{\vec{r}}_{i}(t)-{\left\langle {\vec{r}}_{i}(t)\right\rangle }_{t_{B}}\right]}^{2}\right\rangle }_{t_{A}}{\left\langle {\left[{\vec{r}}_{i}-{\left\langle {\vec{r}}_{i}\right\rangle }_{t_{A}}\right]}^{2}\right\rangle }_{t_{B}}}/\sigma$ (*42*), where *t_A_* and *t_B_* represent the time intervals [*t* − *t*_0_/2, *t*] and [*t*, *t* + *t*_0_/2], respectively. *DW* and *p*_h_ reflect the short-time dynamics, while τ reflects the long time scale for structural relaxation.

We measure log(τ), log(*DW*), and log(*p*_h_), because they are commonly used in glass studies (*35*, *43*). Using {ρ, *s*_2_} or {log(ρ), log (∣*s*_2_∣)} yields similar results (fig. S15 and movie S4). All the five parameters are normalized (denoted by ~) from 0 (vapor) to 1 (bulk glass) for comparison (Materials and Methods).

### Slow temperature change

*T*_g_ is quasistatically approached by decreasing temperature at Δ*T* = 0.2°C per step with 1 to 3 hours of equilibration at each step. Thus, the observed phenomena are equilibrium features near the surface. *N* quantities may saturate to their bulk values at different depths, which define *N* layers near the surface. All the structural parameters {$\tilde{\rho }$, ${\tilde{s}}_{2}$} saturate to their bulk values at *y*_1_, and all the long-time [$\tilde{\log(\tau )}$] and short-time {$\tilde{\log(DW)},\tilde{\log(p_{h})}$} dynamic parameters saturate at *y*_2_ (Fig. 2E). Their profiles define a dense vapor layer at $5\%<\tilde{\rho }<50\%$ with thickness *y*_0_ − *y_s_*, a surface liquid layer at $50\%<\tilde{\rho }<95\%$ with thickness *d*_1_ = *y*_1_ − *y*_0_, and an intermediate glassy layer at $\tilde{\rho }\geq 95\%$ and $\tilde{\log(DW)}<95\%$ with thickness *d*_2_ − *d*_1_ = *y*_2_ − *y*_1_ (see *y*_*s*,0,1,2_ defined in Fig. 2E). Similar double surface layers with an unexpectedly large thicknesses of more than 100 particles have recently been observed in glasses (*27*, *44*).

**Fig. 3.**
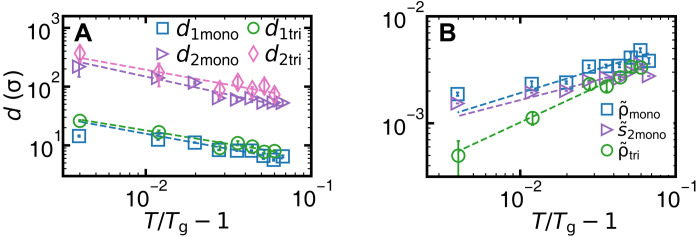
Power laws of surface premelting under slow temperature change. (**A**) Layer thicknesses *d*_1,2_ fitted by Eq. 2 (dashed lines) with *T*_g_ = 25.3°C, α_1_ = 0.51 ± 0.09, α_2_ = 0.61 ± 0.10 for the monolayer and *T*_g_ = 25.0°C, α_1_ = 0.46 ± 0.10, α_2_ = 0.51 ± 0.12 for the trilayer. (**B**) ${\tilde{\rho }}_{y=y_{s}^{\prime }}(T)\;\mathrm{and}\;{\tilde{s}}_{2,y=y_{s}^{\prime }}(T)$ fitted by Eq. 7 with β_ρ_ = 0.45 ± 0.10 and β_*s*_2__ = 0.38 ± 0.08 for the monolayer and β_ρ_ = 0.68 ± 0.09 for the trilayer. The subscript “$y=y_{s}^{\prime }$” represents the parameter at the vapor interface $y_{s}^{\prime }$ defined by Landau theory (section S3). The subscripts “mono” and “tri” in legends represent the monolayer glass and the trilayer glass, respectively. Note that decreasing *T* increases the effective temperature.

*d*_1,2_ represents the penetration depths of the surface effect on structure and dynamics, respectively. Figure 3A shows

$$d_{1,2}\propto {\left(T/T_{g1,2}-1\right)}^{\alpha_{1,2}}$$ (2)

The fitted *T*_g1_ = *T*_g2_ = 25.3°C is consistent with the directly observed complete bulk melting at 25.3°C (movie S2). Moreover, the bulk density jumps and elastic moduli approach zero at 25.3°C (fig. S14), similar to operationally defined glass transition temperature at the sudden change in thermal expansion, shear modulus, or other properties under heating or cooling (*2*). Note that *T*_g_ is derived from *d*_1,2_ via Eq. 2, which circumvents the ambiguity of distinguishing supercooled liquid and glass. The effective temperature linearly decreases with the sample temperature (Fig. 1B), i.e., $\left(T_{g}^{\mathrm{eff}}-T^{\mathrm{eff}})\propto (T-T_{g}\right)$; thus, (*T* − *T*_g_)^α^ in Eq. 2 can be expressed as ${\left(T_{g}^{\mathrm{eff}}-T^{\mathrm{eff}}\right)}^{\alpha }$, which maintains the power-law relationship. Different choices of the threshold values (e.g., 95 or 99.5%) only shift *d*_1,2_ by a constant factor and do not affect the fitted values of *T*_g_ and α (fig. S22).

In crystal premelting, the power-law growth of the surface liquid thickness has been predicted in theory (*15*, *45*) and observed in experiments (*15*, *23*) and simulations (*15*, *46*). Here, we find that it also holds for the two layers of the colloidal glass before reaching *T*_g_ (Fig. 3A). Premelting has only been discussed for crystals at γ_vc_ > γ_vl_ + γ_lc_. γ is the interfacial energy. The subscripts “vc,” “vl,” and “lc” represent the vapor-crystal, vapor-liquid, and liquid-crystal interfaces, respectively. Although double layers have not been reported in premelting, they are theoretically possible when γ_vg_ > γ_vl_ + γ_lg_ > γ_vl_ + γ_ls_ + γ_sg_, i.e., forming a liquid (l) layer and a surface glassy (s) layer between vapor (v) and bulk glass (g).

**Fig. 4.**
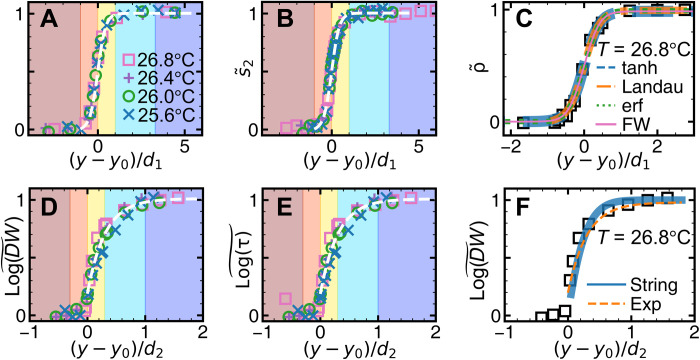
Profiles perpendicular to the surface of monolayer glass under slow temperature change. $\tilde{\rho }$ profiles (**A**) and ${\tilde{s}}_{2}$ profiles (**B**) at different temperatures collapse onto Eq. 3 (white dashed curves). (**C**) The profile of $\tilde{\rho }$ at 26.8°C fitted by hyperbolic tangent function (Eq. 3), the prediction of Landau theory of crystal premelting (eq. S7), error function (erf; eq. S10), and Fisk-Widom (FW) function (eq. S11). $\tilde{\mathrm{Log}(DW)}$ profiles (**D**) and $\tilde{\log(\tau )}$ profiles (**E**) at different temperatures collapse onto Eq. 4 (white dashed curves). (**F**) $\tilde{\mathrm{Log}(DW)}$profile at 26.8°C fitted by exponential function (Eq. 4) and the prediction of cooperative string model (Eq. 5). The colored regions are labeled in Fig. 2E.

In addition, the profiles of structural parameters, $\tilde{\rho }(y)$ and ${\tilde{s}}_{2}(y)$, at different *T* collapse onto a master curve by rescaling *y* with *d*_1_(*T*) (Fig. 4, A and B) and can be fitted by a hyperbolic tangent function

$$\tilde{\rho }(y),{\tilde{s}}_{2}(y)=\{1+\tanh[3(y-y_{0})/2d_{1}]\}/2$$ (3)

The hyperbolic tangent profile commonly exists on vapor-liquid interfaces (*47*), liquid-solid interfaces (*48*, *49*), vapor-solid interfaces (*50*), and solid-solid interfaces (*51*). In addition, error function (erf), the prediction of Landau theory for crystal premelting (*45*), and the Fisk-Widom (FW) function for the interface between fluid phases can fit $\tilde{\rho }(y)\;\mathrm{and}\;{\tilde{s}}_{2}(y)$ equally well (Fig. 4C), because they are in the range of [0, 1] with similar centrosymmetric shapes. The profiles of the dynamic parameters {$\tilde{\log[DW(y)]}$, $\tilde{\log[\tau (y)]}$, and $\tilde{\log[p_{h}(y)]}$} collapse onto a master curve after *y* is rescaled by *d*_2_ (Fig. 4, D and E). They are not centrosymmetric and cannot be fitted by Eq. 3. Instead, they can be fitted by both Eqs. 3 and 4

$$f(y)=1-\exp[-3(y-y_{0})/d_{2}]$$ (4)

$$f(y/d_{2})=f(y^{\prime })=f(y^{\prime }/\sqrt{2})=\mathrm{erf}(y^{\prime })+2y^{\prime }\exp(-{y^{\prime }}^{2})/\sqrt{\pi }-2{y^{\prime }}^{2}[1-\mathrm{erf}(y^{\prime })]$$ (5)

where *f* can represent $\tilde{\log(\tau )},\;\tilde{\log(DW)}$, and $\tilde{\log(p_{h})}$. Equation 4 has also been observed in the surface relaxation of polymer glasses (*52*, *53*) and a dynamic facilitated lattice model of glass (*54*). It implies that the energy barrier height increases with the depth *y* exponentially (*53*). Equation 5 is a prediction about log(τ) in the cooperative string model for the glass surface mobile layer (*55*). We find that Eq. 5 has a very similar shape to Eq. 4 and can also well fit the profiles in Fig. 4F.

### Relationship between local structure and dynamics

**Fig. 5.**
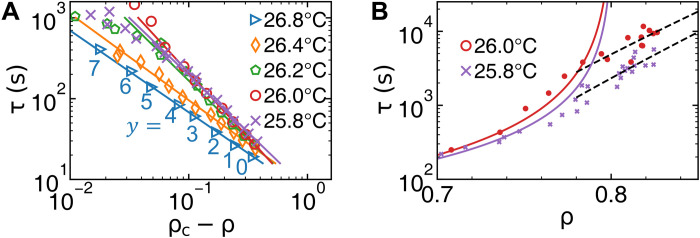
τ(*y*) versus ρ_c_−ρ(*y*) fitted with Eq. 6. (**A**) The fitted ρ_c_ = 0.80 is robust to different temperatures. The depth *y* is labeled at each data point at *T* = 26.8°C; the data points at other *T* have similar depths. (**B**) τ(ρ) follow the mode-coupling transition behavior Eq. 6 (solid curve) at low densities and the Arrhenius behavior log[τ(*y*)] ∝ ρ(*y*) (dashed line) at high densities.

Supercooled liquids generally follow the mode-coupling relation τ(*T*) ∝ [ρ_c_ − ρ(*T*)]^−γ^ (*56*). We observe a similar relation

$$\tau (y)\propto {\left[\rho_{c}-\rho (y)\right]}^{-\gamma }$$ (6)

holds near the glass surface at different depths in contrast to different temperatures in the traditional mode-coupling relation (Fig. 5A). The fitted ρ_c_ = 0.80 is robust to different *T* and agrees with the mode-coupling transition point of two-dimensional (2D) binary glasses measured in simulations (*57*, *58*) and experiments (*26*, *59*). The fitted ρ_c_ = 0.80 is smaller than the nonmelted bulk density (≥0.81), which further confirms the premelting. Equation 6 should hold between any dynamic and structural parameters {e.g., *DW*(*y*) ∝ [*s*_2*c*_ − *s*_2_(*y*)]^−γ^} because their profiles collapse (Fig. 4). Furthermore, the low-density region near the surface exhibits the mode-coupling transition behavior of fragile glass, while the high-density region near the bulk exhibits the Arrhenius behavior of strong glass (Fig. 5B). The similar fragile-to-strong crossover as temperature decreases is observed in water (*60*), metallic glasses (*61*), and organic and inorganic glasses (*62*). Structural dynamic correlation is a research focus in the studies of bulk glass and supercooled liquid (*63*); Fig. 5 provides such a connection near the surface.

Figure 3B shows that the structural parameters at the vapor interface vary with temperature in a power law as predicted by Landau theory of crystal premelting (*45*)

$${\tilde{\rho }}_{y=y_{s}^{\prime }}(T),{\tilde{s}}_{2,y=y_{s}^{\prime }}(T)\propto {\left(T/T_{g}-1\right)}^{\beta_{\rho ,s_{2}}}$$ (7)

Similar to Eq. 2, the power law of Eq. 7 holds after replacing *T* with *T*^eff^. In Landau theory, such power law generally holds for an order parameter near the surface. It has been observed for the sixfold bond-orientational order parameter ψ_6_ (*23*) and magnetism (*64*) in crystal premelting but not for ρ and *s*_2_ in crystal or any order parameters in glass before. Figure 3B and fig. S20B show that ρ and *s*_2_ satisfy this power law in monolayer and multilayer glasses. The vapor interface $y_{s}^{\prime }$ defined by minimizing the free energy in Landau theory for crystal premelting (*45*) is close to *y*_s_ defined by $\tilde{\rho }=5\%$ (fig. S22).

### Multilayer

Monolayer and bilayer colloidal crystals exhibit distinct surface premelting and melting behaviors (*23*), probably because 2D is the critical dimension in which systems have strong long-wavelength fluctuations according to the Mermin-Wagner theorem. However, we find that monolayer and multilayer colloidal glasses have similar behaviors in premelting and melting. This is in accordance with the fact that the results based on *DW* and *DW*_cr_ are similar for monolayer samples. The images of multilayer samples are blurry (fig. S17), so we only measure $\tilde{\rho }$ and $\tilde{\log(DW)}$from the coarse-grained pixel brightness and its fluctuation, respectively (section S2.7). Similar to monolayer samples, Eq. 2 for *d*_1,2_ and Eq. 7 for surface density hold for the bilayer (fig. S20) and trilayer (Fig. 3, A and B) samples. Their structural and dynamic profiles fit Eqs. 3 and 4, respectively (fig. S19).

### Fast temperature change

Crystal melting is usually studied by abruptly increasing the temperature above *T*_m_. Similarly, we abruptly change the temperature controller from 26.5° to 23.3°C across *T*_g_, so that both premelting and melting processes can be studied. *T*(*t*) decreases at a rate of about 10^−3^°C/s (fig. S4).

**Fig. 6.**
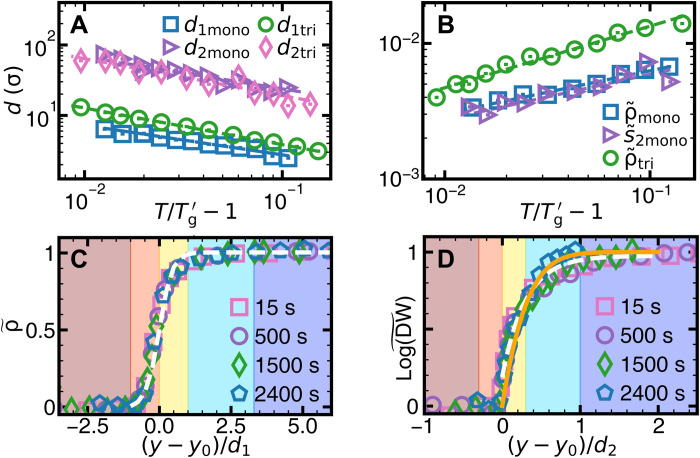
Surface premelting under fast temperature change. (**A**) Layer thicknesses $d_{1,2}(T)\propto {\left(T/T_{g}^{\prime }-1\right)}^{-\alpha_{1,2}}$ (dashed lines) with the fitted $T_{g}^{\prime }=23.4{}^{\circ }C,$ $\alpha_{1}=0.42,\alpha_{2}=0.45$ for the monolayer and $T_{g}^{\prime }=24.5{}^{\circ }C,\alpha_{1}=0.52,\alpha_{2}=0.52$ for the trilayer. (**B**) ${\tilde{\rho }}_{y=y_{s}^{\prime }}(T)$ and ${\tilde{s}}_{2,y=y_{s}^{\prime }}(T)\propto {\left(T/T_{g}^{\prime }-1\right)}^{\beta_{\rho ,s_{2}}}$ (dashed lines) with the fitted β_ρ_ = 0.32 and β_*s*_2__ = 0.33 for the monolayer and β_ρ_ = 0.45 for the trilayer. The errors are smaller than the symbols. (**C**) $\tilde{\rho }$ at different times collapse onto Eq. 3 (white dashed curve). (**D**) $\tilde{\mathrm{Log}(DW)}$at different times collapse onto Eqs. 4 (white dashed curve) and 5 (orange solid curve). *y*_0,*t*=0_ ≡ 0. The colored regions are labeled in Fig. 2E.

Similar to the slow temperature change, *d*_1,2_(*T*) under the fast temperature change fits Eq. 2 with $T_{g1}^{\prime }=T_{g2}^{\prime }=23.4$°C (Fig. 6A); ${\tilde{\rho }}_{y=y_{s}^{\prime }}(T)$ and ${\tilde{s}}_{2,y=y_{s}^{\prime }}(T)$ fit Eq. 7 (Fig. 6B). $T_{g}^{\prime }$ under the fast temperature change is lower (i.e., attraction weaker and effective temperature higher) than *T*_g_ = 25.3°C under the slow temperature change, in accordance with the high *T*_g_ under rapid quenching in vitrification (*3*). The profiles of the structural and dynamic parameters at different times collapse onto two master curves of Eqs. 3 (Fig. 6C) as well as 4 and 5 (Fig. 6D), respectively. Bilayer and trilayer glasses under the fast temperature change also exhibit similar premelting behaviors (Fig. 6 and figs. S19 and S20).

**Fig. 7.**
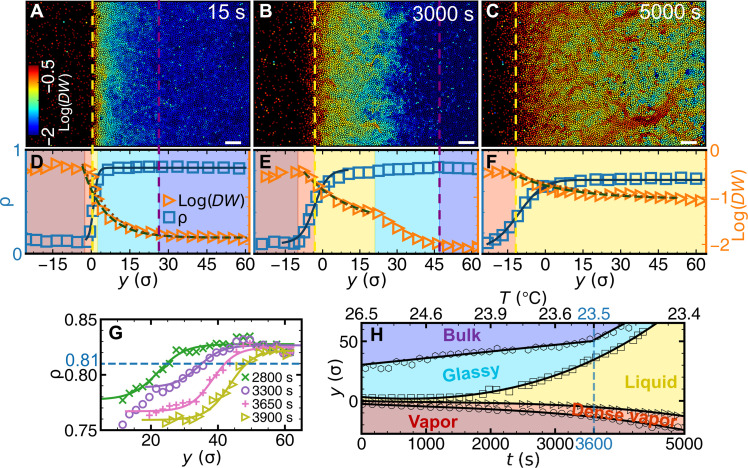
Surface melting under the fast temperature change. (**A** to **C**) The monolayer sample colored by log(*DW*) at different times (also see movie S3). Scale bars, 20 μm. (**D** to **F**) ρ(*y*) and log[*DW*(*y*)] of (A) to (C) fitted by Eqs. 3 (solid curves) as well as 4 (dashed curves) and 5 (dotted curves), respectively (also see movie S4). They share the same double *y* axes. The colored regions are labeled in (H). (**G**) Density profiles across the glassy layer at different times fitted with Eq. 3 (solid curves). (**H**) Evolution of the surface layers.

During the melting stage (Fig. 7), ρ(*y*) and log[*DW*(*y*)] at each instance still follow Eqs. 3 (Fig. 7, D to G) as well as 4 and 5 (Fig. 7, D to F, and fig. S16), respectively. At *t* ≥ 3600 s, the thickness of the glassy layer reaches a constant value (Fig. 7H). The glassy layer can be viewed as the melting front with a hyperbolic tangent density profile (Fig. 7G) and propagates at a constant speed at *t* ≥ 3600 s (Fig. 7H). These behaviors agree with the predictions in Landau theory (*65*) and observations in crystal melting experiment (*49*). The constant speed of melting front has also been observed in ultrastable glass melting (*8*, *16*), whereas the constant width of the melting front has only been conjectured in ultrastable glass without an experimental test (*10*). Overlap function has been used to define the glass melting interface in simulation (*8*). We find that it coincides with the interface defined by $\tilde{\rho }=95\%$ (fig. S12).

### Cooperative rearrangement regions

**Fig. 8.**
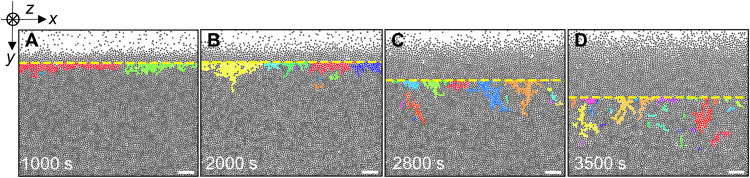
CRRs on the surface for monolayer glass under fast temperature change. (**A** to **D**) Different cooperative rearrangement regions (CRRs) in the monolayer are labeled by different colors at 1000, 2000, 2800, and 3500 s. Scale bars, 20 μm.

Cooperative rearrangement regions (CRRs) are crucial to glass relaxation (*66*), but the observation of individual CRRs requires single-particle kinetics. We measure CRRs near the surface (Fig. 8 and movie S3). CRRs are defined as clusters composed of at least two mobile particles, i.e., top 10% highest DW particles (*26*, *67*). Particles near the vapor interface move rapidly and much less cooperatively; thus, CRRs are not defined, or the whole region would be one huge CRR. CRRs are rare in the bulk. We measure CRRs in (*y*_1_ + *y*_0_)/2 < *y* < *y*_2_ (Fig. 8).

**Fig. 9.**
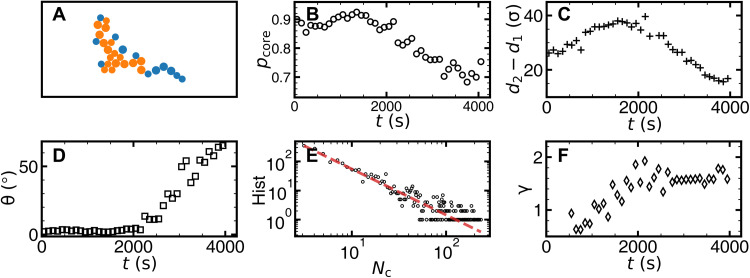
CRR properties for the monolayer sample. (**A**) An example of CRR with core-like particles (orange) and string-like particles (blue). (**B**) Evolution of the fraction of core-like particles in CRRs. (**C**) Evolution of the glassy layer thickness *d*_2_ − *d*_1_. (**D**) The orientation θ of CRR’s long axis relative to the glass surface. θ is weighted average by CRR size and aspect ratio. (**E**) The histogram (hist) of CRR size *N*_c_ during [3000 s, 3100 s] fitted by the power law (dashed red line) with the exponent γ = 1.58. (**F**) γ(*t*) from the size distributions of CRRs. Each data point is averaged over the time interval [*t* − 50 s, *t* + 50 s]. Note that the effective temperature increases with *t*, because the real temperature decreases with *t*.

By assuming that a CRR contains a compact core surrounded by a ramified string-like shell (*66*), the morphology of CRR, i.e., string-like or compact (Fig. 9A), can be described by the fraction of core-like particles *p*_core_ (Materials and Methods) (*26*). The fraction of core-like particles *p*_core_ reaches the maximum at 1800 s (Fig. 9B), indicating that the CRR morphology changes from string-like to compact near 1800 s and to string-like thereafter. This morphological change from compact to string-like as effective temperature increases has been predicted (*66*) and observed (*26*) in bulk glasses. Similarly, the glassy layer thickness (*d*_2_ − *d*_1_)(*t*) shows nonmonotonic behavior and peaks at 1800 s (Fig. 9C). This finding is similar to the observed nonmonotonic dynamic correlation length scale of glasses near a pinned wall (*25*, *26*) or free surface (*68*) accompanied with a similar morphological change of CRRs.

In addition, the major principle axis of the moment of inertia of CRRs changes from parallel to nearly perpendicular to the glass surface at *t* > 1800 s (Fig. 9D), indicating the direction change of CRRs. Such polarized CRRs normal to the surface reflect the transportation of free volumes from surface to bulk, which facilitates the melting (movie S3). In the reverse glass growth via vapor deposition, CRRs were observed to have lower area fractions, i.e., more free volumes, and move from bulk to surface (*27*). These CRRs are similarly elongated normal to the free surface (*27*).

The size distribution of CRRs can be fitted by a power law with exponent γ (Fig. 9E). As the effective temperature increases, γ increases from 0.6 to 1.8, suggesting an increasing fraction of small CRRs. γ reaches a plateau at *t* > 2000 s (Fig. 9F). The measured γ is in the range of [0.5, 2.5] for the power-law exponents of the probability distributions of earthquake magnitudes, i.e., the Gutenberg-Richter law (*69*).

## DISCUSSION

The in situ observations with single-particle kinetics reveal two surface layers. The liquid layer on the surface is stable at a fixed temperature rather than propagating into the bulk, i.e., premelting rather than melting. Note that such identification of premelting does not need the measurement of *T*_g_ or any similarity to crystal premelting. Moreover, glass premelting and melting are similar to those of crystals as shown in Table 1, suggesting that premelting and melting theories for crystals could be generalized to glasses. Ordinary glasses exhibit nucleation-like bulk melting similar to crystal melting, which has been suggested as a support of the thermodynamic origin of glass transition (*7*, *9*, *11*). Similarly, because crystal surface premelting is a thermodynamic phenomenon, the similarities between glass and crystal premelting suggest a thermodynamic origin of glass surface premetling. The study of glass surface melting is at the preliminary stage, which lacks theory and experiment at the single-particle level. Simulations mainly focus on the melting front speed and the crossover depth from surface to bulk melting (*8*, *12*–*14*), while glass surface premelting has not been discussed.

**Table 1.** Comparison between crystals and the colloidal glasses in premelting and melting.

Similarities between crystals and glasses		Only observed in glasses
Premelting ⟹ stable liquid layer	Melting ⟹ propagating melting front	Additional glassy layer
Thickness *d*_1_ ∝ (*T*/*T*_g_ − 1)^α_1_^ (Eq. 2 and Figs. 3 and 6)	Constant speed and width of melting front (Fig. 7)	Thickness *d*_2_ ∝ (*T*/*T*_g_ − 1)^α_2_^ (Eq. 2 and Figs. 3 and 6)
Centrosymmetric profiles of structural parameters [e.g., $\tilde{\rho }(y)$ and ${\tilde{s}}_{2}(y)$; Eq. 3 and Figs. 4 and 6]	Centrosymmetric profiles of structural parameters [e.g., $\tilde{\rho }(y)$ and ${\tilde{s}}_{2}(y)$; Eq. 3 and Fig. 7]	Noncentrosymmetric profiles of dynamic parameters {e.g., $\tilde{\log[DW(y)]}$; Eqs. 4 and 5 as well as Figs. 4 and 6}
Structural parameters at vapor interface ${\tilde{\rho }}_{y=y_{s}^{\prime }},{\tilde{s}}_{2,y=y_{s}^{\prime }}\propto {\left(T/T_{g}-1\right)}^{\beta_{\rho ,s_{2}}}$ (Eq. 7 and Figs. 3 and 6)	\	Dynamic parameters at vapor interface do not follow Eq. 7

Melting is always easier from a free surface than within bulk (section S5). Ultrastable glasses usually melt from surface, which preempts bulk melting, while ordinary glasses usually melt from both surface and bulk. Because bulk region is much larger than surfaces, melting of ordinary glasses is dominated by the bulk. Nevertheless, ordinary glasses have no qualitative distinctions with ultrastable glasses (*70*, *71*) and thus should exhibit similar premelting behaviors, although their surface melting can be easily interrupted by bulk melting. Therefore, a glass with relatively high stability is required for the study of surface melting.

Besides melting, surface mobile layer is an active topic studied in molecular (*5*), metallic (*18*), and polymer glasses (*19*–*22*), especially for thin films. Whether it can be viewed as premelting has not been discussed. The thickness of the surface mobile layer for short-chain polymer glasses changes with temperature in a power law similar to Eq. 2 for crystal premelting but in a linear relation for long-chain polymer glasses (*72*). These suggest that the intensively studied surface mobile layer may be viewed as premelting for short-chain polymer glasses, but probably not for long-chain polymer glasses, because part of the long chains deep under the surface are entangled and frozen (*73*).

In contrast to crystal premelting, the glass surface exhibits an additional glassy layer defined by dynamic parameters, which is beyond the premelting theory. This layer exhibits the above surface liquid layer’s behavior of Eq. 2 (Figs. 3 and 6) but not Eqs. 3 and 7 (Figs. 4 and 6) as listed in Table 1. Furthermore, an interesting structural dynamic correlation analogous to mode-coupling equation is observed near the surface, but it varies with depth rather than with temperature in the mode-coupling relation (Fig. 5).

In addition, the CRR morphology evolution under fast heating is measured. The CRR morphology changes from compact to string-like particles, which is accompanied with the nonmonotonic surface glassy layer thickness change (Fig. 7H), similar to the CRR morphology changes observed in bulk accompanied with a nonmonotonic change of dynamic correlation length in (*25*, *26*). CRRs propagate toward the bulk, and thus they are elongated normal to the free surface in deeper regions. CRRs have slightly lower local densities, and therefore they bring free volumes into the bulk to facilitate the melting. This is similar to the opposite propagations of CRRs (from bulk toward surface) in a reverse process (vapor deposition growth of the glass) in (*27*).

Monolayer and multilayer glasses exhibit similar premelting and melting behaviors, indicating that the dimensionality effect is not prominent for glasses. This phenomenon is in accordance with the similar behaviors for 2D and 3D bulk glasses (*40*, *41*) and in contrast to the distinct premelting and melting behaviors for monolayer and bilayer crystals (*23*, *46*).

## MATERIALS AND METHODS

### Interaction between colloidal spheres

Nonfluorescent liquid dye (D98010 Chromatint jet black 1990, Chromatech Incorporated) is heated at 75°C for 7 hours and sonicated for 1 min. It is then added, 23% by volume, into the aqueous PMMA colloidal suspension (PAMMA-R, microParticles GmbH) with 30% volume fraction of PMMA spheres. The dye induces a depletion-like attraction among PMMA spheres, because its strength increases with the dye concentration and the measured attraction agrees with the Oosawa-Asakura model of depletion attraction (*23*). Without the dye, PMMA spheres exhibit no attraction.

We measure the radial distribution function $g(r)=\frac{1}{n^{2}}\langle \rho ({\vec{r}}^{\prime }+\vec{r},t)\rho ({\vec{r}}^{\prime },t)\rangle$ of a dilute monolayer of monodispersed PMMA spheres at equilibrium (fig. S1A) to calculate their pair interaction (Fig. 1 and fig. S1B) (*74*). $\vec{r}$ is particle position, $\rho (\vec{r},t)=\sum_{j=1}^{N(t)}\delta [\vec{r}-{\vec{r}}_{j}(t)]$, *n* = *N*/*A* is the area density in a field of view containing *N* particles on average, and 〈 〉 is the average over angles and time. Note that the small effects of the diffraction rings in the bright-field image analysis (*75*) and polydispersity have been corrected in the measurement of *g*(*r*). According to the Ornstein-Zernike integral equation in the liquid structure theory (*74*, *75*), *U*(*r*) is calculated from *g*(*r*) by either the Percus-Yevick (PY) approximation or hypernetted-chain (HNC) approximation (*76*)

$$U(r)=-k_{B}T\ln[g(r)]+\left\{ \begin{array}{cc} k_{B}TnI(r) & \;(\mathrm{HNC})\\ k_{B}T\ln[1+nI(r)] & \;(\mathrm{PY}) \end{array}\right.$$ (8)

where $I(r)=\int_{A}[g(r^{\prime })-1-nI(r)][g(|{\vec{r}}^{\prime }-\vec{r}|)-1]d^{2}r^{\prime }$. PY and HNC are more accurate for hard and soft potentials, respectively. They are accurate only for low-density gas phase at equilibrium, and the iteration in the algorithm converges at area fractions below 15%. Thus, our area fractions are about 10% for the samples of the *U*(*r*) measurements. *U*(*r*) from the two approximations is highly consistent, suggesting that the calculation is reliable. This algorithm assumes that *U*(*r*) is pairwise additive, which may not exactly hold in dense colloids. Nevertheless, the measured *U*(*r*) describes that the attraction is tunable. The attraction strength *U*_min_, i.e., the minimum of *U*(*r*), is at *r* = 2.6 and 3.4 μm for small and large particles, respectively (fig. S1B). Thus, the attraction range is 0.5 μm for both particles, which is robust under different temperatures. Figure S1B shows that the effective temperature *T*_eff_ = *k*_B_*T*/∣*U*_min_(*T*)∣ linearly decreases with *T*.

### Sample preparation

Binary PMMA spheres are used to avoid crystallization. The number ratio of large particles to small particles ranges from 40:60 to 60:40 in all the 10 samples of monolayer, bilayer, and trilayer glasses. Each kind of sphere has a small polydispersity of 2.2%. The sample preparation process is schematically shown in fig. S2. A colloid droplet is placed on a glass coverslip and covered by another coverslip (fig. S2A). We use coverslips instead of glass slides, because rigorously cleaned coverslips by deionized water and flame can effectively avoid particle sticking. The sample is sealed by epoxy to fix the sample thickness (fig. S2B). The sample thickness can be controlled by the volume of the colloid droplet, e.g., 1.5 μl for the monolayer sample. The colloidal droplet is spread in the center of 22 × 22 mm^2^ area of the coverslip due to capillary force. Although the two glass plates are not perfectly parallel in the whole centimeter-sized sample area, they are sufficiently parallel in the millimeter-sized region such that a monolayer (or bilayer or trilayer) can form in a millimeter-sized area. Figure S3 shows a schematic of a trilayer sample. The *x* and *y* directions are along and perpendicular to the surface of the colloidal glass, respectively. The *z* direction is perpendicular to the coverslips. Surface layers in the main text are along the *y* direction, whereas monolayer, bilayer, and trilayer samples refer to the number of layers in the *z* direction (fig. S3).

To make the colloid droplet spread into a monolayer, the initial particle density needs to be low. Thus, colloidal particles are in a vapor phase in a freshly made sample. To form a glass, the sample is tilted in gravity under the room temperature, such that particles sediment to the edge with the desired wall separation and form a dense liquid or glass (fig. S2C). The sample is then placed horizontally and relaxed for 1 day to dense liquid and vapor under the room temperature *T* = 23°C where particles have negligible attractions (fig. S2D). By increasing the sample temperature, i.e., enhancing the attraction, to the desired initial temperature for measurements, the liquid region starts to vitrify into a glass and continues to grow through the slow layer-by-layer vapor deposition for more than 10 hours until the vapor phase is nearly depleted (fig. S2, E and F, and movie S1). The measurement is under quasi-equilibrium condition after a long-time equilibration without gravity. Sedimentation is commonly used for colloidal crystal fabrication and bulk studies, while the surface studies are limited and during the sedimentation processes for glasses (*27*) and crystals (*77*).

### Temperature change

The objective heater (Bioptechs) with a 0.1°C resolution heats a sample area of a circle with a diameter of 10 mm. The temperature gradient at the edge of the heated region can pump the ambient dye into the heated region via thermophoresis, thereby enhancing the attraction. For the slow temperature change, *T* decreases at 0.2°C per step, and the sample is equilibrated for 1 to 3 hours at each step. For the fast temperature change, although the temperature controller is abruptly set across the glass transition temperature, the measured sample temperature decreases at a rate of about 10^−3^ to 10^−2^°C/s because of the slow heat conduction of the objective, coverslips, and air (see fig. S4).

### Relaxation time

The structural relaxation time τ is measured from the decay time of the self-intermediate scattering function *F_q_*(*t*) (fig. S9). At depth *y*, *F_q_*(*y*) is defined as (*78*)

$$F_{q}(y,t)=\langle e^{i\vec{q}\cdot [{\vec{r}}_{i}(t_{0}+t)-{\vec{r}}_{i}(t_{0})]}\rangle_{t_{0},i}$$ (9)

where ${\vec{r}}_{i}(t)$ is the position of particle *i* at time *t*, $\vec{q}$ is the first peak position of the structure factor of the bulk glass, and 〈 〉_*t*_0_,*i*_ denotes the ensemble average over particles in the stripe at *y* and at the initial *t*_0_. *F_q_*(*t*) reflects the particles’ motions at lag time *t* on a length scale 2π/*q* and can be directly measured in the scattering experiment (*78*). *F_q_*(*t*) at different *y* can be fitted by *F_q_*(*t*) ∼ *e*^−(*t*/τ)^β^^ with the relaxation time τ(*y*).

### Local structural entropy

For a binary system (*79*), the two-point structural entropy $s_{2i}=-\frac{1}{2}\sum_{\nu }\rho_{\nu }\int d\vec{r}\{g_{\nu i}(\vec{r})\ln[g_{\nu i}(\vec{r})]-[g_{\nu i}(\vec{r})-1]\}$, where ρ_ν_ is the local number density of large or small particles, ν represents large or small particle, and $g_{\nu i}(\vec{r})$ is the radial distribution function of particle *i*. We choose the cutoff distance *r* = 2.5 σ for the integration, because *g*(*r* > 2.5 σ) ≃ 1, and thus the long-ranged part barely affects *s*_2_ (*36*).

### Mean square displacement

Particle dynamics are often characterized by the mean square displacement (MSD)

$$\mathrm{MSD}(t)=\langle \text{Δ}r^{2}(t)\rangle =\langle {\left[{\vec{r}}_{i}(t^{\prime }+t)-{\vec{r}}_{i}(t^{\prime })\right]}^{2}\rangle$$ (10)

where 〈 〉 is the average over time and particles in the chosen region. The measured MSD in fig. S10 shows that the out-of-cage time is about 30 s for particles near the surface (*y* = 10 σ).

### Cage-relative Debye-Waller factor

Besides the Debye-Waller factor, we also calculate the cage-relative Debye-Waller factor (*39*)

$${DW}_{\mathrm{cr}i}(t)=\sqrt{\left\langle {\left[{\vec{r}}_{i}^{\prime }(t)-\langle {\vec{r}}_{i}^{\prime }(t)\rangle \right]}^{2}\right\rangle }/\sigma$$ (11)

where $\left\langle {\vec{r}}_{i}^{\prime }(t)\right\rangle$ is the average position of particle *i* during [*t* − *t*_0_/2, *t* + *t*_0_/2]. It is different from the DW factor measured in the laboratory frame in which each particle’s position is described in its local frame defined by the mean position of its *N* nearest neighbors: ${\vec{r}}_{i}^{\prime }\equiv {\vec{r}}_{i}-\sum_{j}{\vec{r}}_{j}/N$. Thus, the effect of long-wavelength fluctuations is excluded in *DW*_cr_.

### Parameter normalization

Log(*DW*) is normalized into [0, 1] as

$$\tilde{\log(DW)}\equiv \frac{\log[DW(y)]-\log{\left(DW\right)}_{v}}{\log{\left(DW\right)}_{b}-\log{\left(DW\right)}_{v}}$$ (12)

where subscripts “b” and “v” represent the values in bulk glass and vapor, respectively. $\tilde{\log[DW(y)]}$ increases with *y*, whereas log[*DW*(*y*)] decreases with *y*, because log(*DW*)_b_ − log (*DW*)_v_ < 0. Other parameters are similarly normalized.

### Cooperative rearrangement regions

The fuzzy sphere model assumes that a CRR contains a compact core surrounded by a ramified string-like shell (*66*). The string-like shell and the compact core are predicted (*66*) and observed (*26*) to dominate at low and high temperatures, respectively. A particle is labeled core-like only if it has at least three nearest neighbors in the CRR and at least two of them have at least three nearest neighbors in the CRR; otherwise, it is labeled as string-like particles (Fig. 9) (*26*).

## Supplementary Materials

### This PDF file includes:

Supplementary Text Sections S1 to S5 Figs. S1 to S23 Legends for movies S1 to S4 References
