# Supplementary material for: Surface premelting and melting of colloidal glasses
Source: Sci Adv. 2023 Mar 17;9(11):eadf1101. doi: 10.1126/sciadv.adf1101 (PMC10022898; doi:10.1126/sciadv.adf1101)
Supplement: Supplementary file 1 — Supplementary Text Sections S1 to S5 Figs. S1 to S23 Legends for movies S1 to S4 References [file sciadv.adf1101_sm.pdf]

Science Advances
AAAS

Supplementary Materials for

Surface premelting and melting of colloidal glasses

Qi Zhang *et al.*

Corresponding author: Yilong Han, yilong@ust.hk

Sci. Adv. **9**, eadf1101 (2023)
DOI: 10.1126/sciadv.adf1101

The PDF file includes:

Supplementary Text
Sections S1 to S5
Figs. S1 to S23
Legends for movies S1 to S4
References

Other Supplementary Material for this manuscript includes the following:

Movies S1 to S4

1. Glass stability

Ultrastable glasses have been fabricated in metallic (*80*), molecular (*32*) and polymer (*81*) systems by vapor deposition, but not yet in colloids. Here, by increasing the sample temperature, i.e. enhancing the attraction, the colloidal liquid region starts to vitrify into a glass and continues to grow through the slow layer-by-layer vapor deposition for more than 10 h until the vapor phase is nearly depleted (fig. S2 and movie S1). Such glasses formed by vapor deposition are highly stable due to effective surface relaxation (*32, 82*). $\chi_\mathrm{d} = v_\mathrm{d}\tau_\alpha^\mathrm{s}/\sigma$ is inversely proportional to the degree of surface relaxation which has been proved to be the dominant factor on glass stability (*82*). v_d is the deposition rate, τ_α^s is the relaxation time of the surface layer, and σ is the average particle diameter. A glass formation process with a low χ_d produces a highly stable glass. The time $t_0 = \sigma^2/(2(d_\mathrm{s} - 1)D)$ is the mean time for a particle on the vapor–liquid interface moving one mean diameter, and is commonly used as τ_α^s (*83*). D is the diffusion coefficient of a particle on the surface (*84*), and d_s is the dimension of the sample. In our typical samples, $D = 1.5 \times 10^{-14}$ m^2/s and $v_\mathrm{d} = 6.3 \times 10^{-9}$ m/s (fig. S6); thus, $\chi_\mathrm{d} = v_\mathrm{d}\sigma/(2D) \approx 0.5$. By contrast, N,N'-bis(3-methylphenyl)-N,N'-diphenylbenzidine (TPD) with particle diameter ~ 1 nm (*85*) and $D \sim 10^{-19}$ m^2/s (*86*) can form a common type of ultrastable glass when the vapor deposition rate $v_\mathrm{d} \leq 10^{-8}$ m/s (*87*). Based on these values in the literature, we find that the ultrastable TPD glass can form when $\chi_\mathrm{d} < 100$. $\chi_\mathrm{d} \approx 0.5$ of our colloidal glass falls into this range. Compared with the ballistic motions of small molecules, Brownian motions of large colloidal particles lead to a longer relaxation time and lower deposition rate. These two effects roughly cancel each other, resulting in a relatively small χ value.

Besides the surface relaxation rate, the bulk density of our glasses 0.83 is higher than typical colloidal glasses and close to the upper limit 0.855 of the randomly close-packed hard disks (*88*). Moreover, the bulk elastic moduli in fig. S14 are one order of magnitude higher than those of normal colloidal glasses (*89, 90*) and those of colloidal crystals of the same system (*23*), see the detailed

calculation in Section B.5. In summary, the small $\chi_{\rm d}$, melting from surface instead of bulk, and high elastic moduli suggest that our glasses appear to be relatively stable. On the other hand, it is challenging to measure the ultrastability directly from the relaxation time τ due to our limited experimental time scale.

2. Data analysis

2.1 Image analysis for large and small particles

Particles can be identified by conventional image analysis (*33*). To further distinguish large and small spheres, we measure the radius of gyration of the pixel brightness $R_{\rm g}$ and the total brightness of each particle's image. These two parameters can distinguish large and small particles (fig. S5).

2.2 Dividing the field of view into stripes

The field of view is divided into stripes parallel to the glass surface with a width of 30 pixel $\approx 1\ \sigma$ (fig. S7). We measure various quantities for each particle i and average them in each stripe region to obtain their profiles along the y direction.

2.3 Voronoi tessellation

We use the radical Voronoi tessellation in the Voro++ library (*91*) instead of traditional Voronoi tessellation for our binary system, because otherwise, the bisecting line between large and small spheres may cut through the large sphere and cannot reflect the real Voronoi area associated with each sphere. In the 2D radical Voronoi tessellation, the radical curve is composed of the points with the same tangent length for the two neighboring spheres, thus the tangential lines from the radical curve to each sphere's surface have the same length. Radical Voronoi tessellation avoids the intersection through

spheres and possesses the topological features of the tessellation. Fig. S8 illustrates an example of the radical Voronoi tessellation of our binary system. The local density of particle i, $\rho_i \equiv \pi\sigma_i^2/(4A_i)$, where A_i is the area of its Voronoi polygon.

2.4 Overlap function

Overlap function has been used to define the glass melting interface in simulation (*8*). It reflects the similarity of particles' configurations after a time interval t (*8*):

$$F_{\mathrm{m}}(t, y) = \frac{1}{N_y} \sum_i \prod_{\tau=0}^{t} \Theta(a - |\vec{r}_i(\tau) - \vec{r}_i(0)|) \times \Theta(y - (y_i(0) - 1))\Theta(y_i(0) - y), \tag{S1}$$

where $\Theta(x)$ is the Heaviside step function, N_y is the number of particles in the stripe at y. If a particle moves more than $a = 1.5\ \sigma$, it is deemed mobile and no longer contributes to F_{m} even if it eventually returns close to its original position (*8*). Fig. S12A shows $F_{\mathrm{m}}(t)$ under the fast temperature change. The melting interface is defined as $F_{\mathrm{m}} = e^{-1}$, as shown in fig. S12A (*8*). Interestingly, the melting interface at every instance determined from the dynamic parameter $F_{\mathrm{m}}(t, y)$ (red cross in fig. 12B) coincides with the interface between surface liquid and glassy layer defined by the structural parameter $\tilde{\rho} = 95\%$ in the main text.

2.5 Melting and glass transition temperatures

The glass transition temperature featured with infinite relaxation time is often identified as the mode-coupling critical temperature T_{MCT} measured by extrapolating the relaxation time to infinity using Eq. 5 from mode coupling theory (*56, 92*). The fitted $\rho_{\mathrm{c}} = 0.80$ in Fig. 5 corresponds to the bulk density at 25.4℃ which is close to the melting temperature 25.3℃ for the slow temperature change.

Glass is also empirically defined when the material has become too viscous to flow in a reasonable time scale (*56*), e.g. the viscosity (or relaxation time) $> 10^{15}$ times of that of a normal liquid (*56*). Given that viscosity changes rapidly near the glass transition temperature, T_{g} is not sensitive to the

choice of threshold value. Besides viscosity, properties such as heat capacity, density and elastic moduli suddenly change during cooling or heating at a certain rate (*2*); either the onset, the middle or the ending of such a change has been used as an empirical definition of T_{g} (*17*). In the main text, T_{g} is fitted from Eq. 1 on the penetration depth, which avoids distinguishing supercooled liquid and glass. The fitted T_{g} agrees with the abrupt change in bulk density, the extrapolated zero elastic moduli shown in fig. S14 and the fully melted sample from the direction observation (movie S2).

The elastic moduli in the main text are calculated according to the following four steps (*23, 93*): (1) Construct the covariance matrix of particle displacements and calculate its eigenvalues and eigenvectors. The displacement covariance matrix $C_{ij} = \langle (u_i(t) - \langle u_i(t) \rangle_t)(u_j(t) - \langle u_j(t) \rangle_t) \rangle_t$, where a particle's coordinate $u_i \in x_1, y_1, \ldots, x_N, y_N$. $\langle \rangle_t$ is the average over frames. $i, j = 1, 2, \ldots, 2N$. N is the number of particles. We track the positions of $N \approx 10^3$ particles in the bulk region for 12000 frames at 20 frames/s for measuring the high-frequency modes. We diagonalize the large $2N \times 2N$ matrix C_{ij} to solve the eigenvectors, which are the polarization vectors of the normal modes. The eigenvalues $\lambda = k_{\mathrm{B}}T/(m\omega^2)$, which provide the angular frequencies ω of the corresponding modes. The mass of a small particle m is mass unit. The accurate value of the particle mass is not needed because it is canceled in the final result. The displacement of a particle is in the unit of the average diameter σ. The wave vector $\vec{q}$ is in the unit of $1/\sigma$, and ω is in the unit of $\sqrt{k_{\mathrm{B}}T/m\sigma^2}$. (2) Extract the dispersion relation $\omega(q)$ (fig. S13B). The Fourier decomposition of the eigenmodes into transverse and longitudinal components yields two spectral functions, $E_T(q, \omega)$ and $E_L(q, \omega)$, respectively:

$$E_T(q, \omega_j) = \langle | \sum_{i=1}^{N} [\widehat{q} \cdot \vec{e}_j(i)] \exp(i\vec{q} \cdot \vec{r}_i) |^2 \rangle, \tag{S2}$$

$$E_L(q, \omega_j) = \langle | \sum_{i=1}^{N} [\widehat{q} \times \vec{e}_j(i)] \exp(i\vec{q} \cdot \vec{r}_i) |^2 \rangle, \tag{S3}$$

where $\widehat{q} = \vec{q}/|\vec{q}|$, $\langle \rangle$ is average over different directions of $\vec{q}$, $\vec{e}_j(i) = (e_{ix}, e_{iy})_{\omega_j}$ is the polarization vector of the ith particle corresponding to the jth mode. $\omega(q)$ is obtained by selecting an ω that maximizes E_T or E_L for each fixed $q = |\vec{q}|$ (fig. S13A). As at low q, ω and q exhibit a linear relationship (fig. S13B),

thus we can obtain the longitudinal modulus, $M = \rho_{2D}(\lim_{q\to 0}(\partial\omega_L/\partial q))^2$, the shear modulus $G = \rho_{2D}(\lim_{q\to 0}(\partial\omega_T/\partial q))^2$, and the bulk modulus, $B = M{-}G$. The areal density $\rho_{2D} = 6mh/(\pi\sigma^3)$ and the height of the sample cell $h \approx \sigma$. The bulk and shear moduli are (38000, 18000) $k_\mathrm{B}T/\sigma^2$ for the vapor-deposited glass, which are much higher than (450, 100) $k_\mathrm{B}T/\sigma^2$ for the colloidal glasses (*89, 90*) and (800, 400) $k_\mathrm{B}T/\sigma^2$ for the colloidal crystals (*23*). These results are converted into the same unit for comparison and are in accordance with the fact that stable glasses have larger elastic moduli.

2.6 Distribution profiles in the y direction

Fig. S15A shows that the profiles of structural parameters $\{\tilde{\rho}(y),\ \tilde{s}_2(y)\}$ are similar to their logarithm $\{\log\widetilde{(\rho(y))}, \log\widetilde{(|s_2(y)|)}\}$ because they are rescaled into the same range of [0, 1] and quite centrosymmetric. Thus comparing $\log\widetilde{(DW(y))}$ with $\{(\tilde{\rho}(y),\ \tilde{s}_2(y))\}$ in Fig. 2E or $\{(\log\widetilde{(\rho(y))}, \log\widetilde{(|s_2(y)|)})$ yields similar results (fig. S15, A and B). We use $\log(\tau)$ instead of τ in the main text because $\log(\tau)$ is popularly used in the literature. Consequently, $\log(DW)$ and $\log(p_\mathrm{h})$ are used for consistency.

Similar to the density profile of the glassy layer fitted by Eq. 2 in Fig. 7G of the main text, the profile of the dynamic parameter $\log(DW(y))$ can be fitted by Eqs. 3 and 4 of the main text in the glassy layer at different times as shown in fig. S16.

2.7 Multilayer glasses

For bilayer and trilayer glasses, the bright-field images are blurry because the different refractive indexes of water and PMMA spheres produce diffractions from other layers in the z direction. We find that the dense region is brighter and the pixel brightness fluctuates more at regions with more mobile particles (fig. S17, A and B). Therefore, the pixel brightness (B) and its fluctuation (i.e. the standard deviation of $B(t)$ over 30 s, std(B)) can be used to quantify the coarse-grained local density and dynamics, respectively. This result is confirmed by the calibration in the monolayer sample in fig. S18.

We measure the mean brightness B of each coarse-grained 30×30 pixel^2 region and its $\log(\mathrm{std}(B))$ and then rescale them to [0, 1], denoted as $\tilde{B}$ and $\widetilde{\log(\mathrm{std}(B))}$, respectively. In monolayer samples, their profiles well agree with the profiles of $\tilde{\rho}$ and $\widetilde{\log(DW)}$ respectively (fig. S18, A and B) and produce the same fitted T_g and power law exponent α from $d_{1,2}$ (fig. S18C). Similarly, we measure the coarse-grained $\tilde{B}$ and $\widetilde{\log(\mathrm{std}(B))}$ for the bilayer and trilayer samples. $\tilde{B}(y)$ and $\widetilde{\log(\mathrm{std}(B)(y))}$ profiles can be fitted by Eq. 2 and Eqs. 3, 4 in the main text, respectively. Thus, their profiles at different temperatures collapse after being rescaled by their layer thicknesses (fig. S19). Layer thicknesses $d_{1,2}$ measured from $\tilde{B}(y)$ and $\widetilde{\log(\mathrm{std}(B)(y))}$ follow the power law of Eq. 1 in the main text (Figs. 3A, 6A and fig. S20, A and C). $\tilde{B}$ at the vapor interface also follows the power law of Eq. 6 in the main text (Figs. 3B, 6B and fig. S20, B and D). All these results are similar to those of monolayer samples. Given that multilayer samples only have coarse-grained data, the other parameters $\{s_2,\ p_\mathrm{h},\ \log(\tau)\}$ requiring single-particle resolution cannot be accurately measured. Nevertheless, the measurable structural parameter $\tilde{B}$ and dynamic parameter $\widetilde{\log(\mathrm{std}(B))}$ are sufficient to reach similar conclusions as monolayer glasses. Note that bilayer and trilayer are estimated from small particles, and the exact number of layers in the z direction is not rigorously defined because the structure is amorphous.

3. Landau theory

For a 2D vapor–solid system with a 1D free surface along the x direction, the general form of the Landau free energy functional (*45*) is

$$F\{\psi\} = \int_{y_\mathrm{s}}^{\infty} dy[\delta(y - y_\mathrm{s}) f_\mathrm{s}(\psi) + \frac{1}{2}(\frac{d\psi}{dy})^2 + f(\psi)], \tag{S4}$$

where y_s is the position of the vapor interface, and ψ is a scaler order parameter such as density. The three terms in Eq. S4 represent the interface, the surface region with a gradient and the homogenous bulk region, respectively. The Taylor expansion of the bulk term $f(\psi)$ is (*45*)

$$f(\psi) = \frac{1}{2} a\psi^2 - \frac{1}{4} b\psi^4 + \frac{1}{6} c\psi^6 + \cdots . \tag{S5}$$

Minimising $F\{\psi\}$ by $\delta F/\delta\psi = 0$ yields (*45*)

$$f(\psi) = \frac{1}{2}(\frac{d\psi}{dy})^2 + f(\psi_\mathrm{b}). \tag{S6}$$

Eq. S6 has been solved analytically in ref. (*45*), and we rewrite the solution in the following form (*45*):

$$(y) \sim 1/(Q \coth^2(\sqrt{P} y + S) - \frac{1}{2} \ {}^{2}_{\mathrm{b}})^{\frac{1}{2}}, \tag{S7}$$

where Q, P and S are parameters related to temperature. $P = \frac{1}{2} b\psi_\mathrm{b} - a$; $Q = \frac{3}{2}\psi_\mathrm{b}^2 - \ {}^{*2}_{\mathrm{b}}$; $S = \mathrm{arccoth}((\frac{1+2R/\psi^2(y=y_\mathrm{s})}{1+2R/\psi_\mathrm{b}^2})^{1/2})$; $R = \ {}^{2}_{\mathrm{b}} - \ {}^{*2}_{\mathrm{b}}$; ${}^{*}_{\mathrm{b}} = 2b^2/(9c)$ is the bulk value at the melting point; $\psi_\mathrm{b} = \frac{1}{2} c(b + \sqrt{b^2 - 4ac})$ is the bulk value. ψ_b can be directly measured; therefore, the three free parameters a, b and $\psi(y = y_\mathrm{s})$ can be solved from the fitted Q, P and S from Eq. S7.

Substituting Eq. S6 into Eq. S4, we obtain the free energy of the whole system in Eq. S4 (*45*):

$$F = f_\mathrm{s}(\psi(y = y_\mathrm{s})) + \int_{y_\mathrm{s}}^{\infty} dy[(\frac{d\psi}{dy})^2 + f(\psi_\mathrm{b})]. \tag{S8}$$

Because the last term $\int_{y_\mathrm{s}}^{\infty} f(\psi_\mathrm{b}) dy$ is a constant, we only need to consider the first two terms (*45*)

$$F' = f_\mathrm{s}(\psi(y = y_\mathrm{s})) + \int_{y_\mathrm{s}}^{\infty} dy(\frac{d\psi}{dy})^2. \tag{S9}$$

Under a given vapor interface position y_s, the fitting of Eq. S7 gives a, b, c in Eq. S5. By tuning y_s, we obtain the corresponding F' in fig. S21A. The minimum of F' gives y_s and $\psi(y)$ of the equilibrium state. The bulk energy is shown in fig. S21B with two local minimums corresponding to vapor and glass, respectively. The local maximum separating the vapor region and glass region (red dashed line in fig. S21B) defines the vapor–liquid interface y_0. With y_s and y_0 identified from fig. S21, A and B, respectively, the liquid–glass interface is $y_1 = y_0 + (y_0 - y_s)$ because the density profile is centrosymmetric.

3.1 Different definitions of the surface layers

The three interfaces defined through structural profiles are vapor–dense vapor interface y_s, dense vapor–liquid interface y_0 and liquid–glassy layer interface y_1. In the main text, they are defined as $\tilde{\rho}(y = y_{s,0,1}) = 5\%, 50\%, 95\%$, respectively. Landau theory can provide an alternative definition of $y_{s,0,1}$ as mentioned in the above section and fig. S21. fig. S22 shows that the positions of $y_{s,0,1}$ defined by these two methods are equivalent. Shifting the threshold densities to (5%, 50%, 95%) only shifts the positions of interfaces by a constant prefactor (fig. S22B) and does not influence the fitted T_g and the power law exponents in the main text.

3.2 Interface profiles

The density or other structural parameters usually exhibit a centrosymmetric distribution profile in the normal direction across various interfaces. For example, the profiles can be fitted by the hyperbolic tangent function at vapor–liquid interfaces (*47, 94, 95*), liquid–solid interfaces (*48, 49, 96, 97*), vapor–solid interfaces (*50*) and solid–solid interfaces (*51*). Although refs. (*47–49, 51*) did not provide the fitting, we find that their data can be fitted by the hyperbolic tangent function. The profiles of the

parameter ψ across various interfaces have also been fitted by the error function (*95, 98*),

$$(y) \sim \mathrm{erf}(\sqrt{\pi} y), \tag{S10}$$

and the function proposed by Fisk and Widom (FW) (*99*):

$$(y) \sim \sqrt{2}\tanh(\sqrt{6}y)/(3 - \tanh^2(\sqrt{6}y))^{\frac{1}{2}}. \tag{S11}$$

In addition, the surface profile in crystal premelting has been predicted as Eq. S7 from Landau theory (*45*), but it has not been tested in experiments or simulations. All the four functions (hyperbolic tangent function, error function, FW function and Eq. S7 in Landau theory) are centrosymmetric in [0, 1]; thus, they are similar and can all fit our measured profiles (Fig. 4, A and C).

4. Power law in polymer thin-film glasses

The surface mobile layer is usually studied by comparing the properties of thin-film glasses with different thicknesses (*72*). For example, for polymer thin-film glasses with low molecular weight, their glass transition temperature T_g^* and film thickness d can be empirically fitted as follows (*72*):

$$d \propto (1 - T_g^*/T_g)^{-\delta} \tag{S12}$$

where T_g is the bulk glass transition temperature. The low T_g^* for a thin film is due to the liquid-like surface mobile layer whose thickness increases with temperature and diverges at bulk T_g (*72*). A thin-film glass can be approximately regarded as the surface region of a bulk glass; thus, the empirical Eq. S12 essentially describes the premelting behavior in Eq. 1 of the main text. However, premelting in thin-film or bulk glasses has not been proposed or compared with the Landau prediction of Eq. S7 for crystal premelting.

5. Surface melting in ordinary and ultrastable glasses

Most facets of a crystal's surface exhibit surface premelting (*15*). Such surface liquid can be viewed as a huge postcritical nucleus once the crystal reaches the melting temperature; thus, the crystal melts heterogeneously from free surfaces and preempts liquid nucleation from the interior of the bulk (*100*). Consequently, superheated crystals and homogenous crystal melting rarely exist in nature. By contrast, ordinary glasses melt homogeneously within the bulk, and surface melting is negligible because bulk is much larger than the surface region. Ultrastable glass was discovered in 2007 (*32*) and its surface melting was observed in 2009 (*5*). The surface melting front propagates to the maximum depth l_c where it meets the melted bulk (*8, 9, 16*). Therefore, the surface region within l_c exhibits surface melting and the deep bulk region exhibits homogeneous bulk melting. This unified picture can explain the observed bulk melting in ordinary glasses and surface melting in ultrastable glasses (*8, 10*): If l_c is too small to be observed in a macroscopic experiment, then surface melting cannot be detected. It can be qualitatively expected that the melting behavior depends not only on glass stability but also on heating rate. At sufficiently slow heating, even ordinary glass can melt from the surface, and at sufficiently fast heating, even ultrastable glass can melt from within the bulk.

More quantitatively, the velocity of the melting front v and the bulk glass-to-liquid transformation time τ_{trans} can determine l_c as (*8*):

$$l_c(T) = v(T)\tau_{\text{trans}}(T). \tag{S13}$$

Glass melting experiments (*101, 102*) and simulations (*8, 12*) have revealed the empirical equation

$$v \sim {\tau_\alpha}^{-\gamma}, \tag{S14}$$

where γ is a constant less than 1. The liquid relaxation time τ_α follows the Vogel–Fulcher–Tammann (VFT) equation

$$\tau_\alpha \sim e^{\mathcal{A}/(T-T_0)}, \tag{S15}$$

where T_0 is the ideal glass transition temperature. $\mathcal{A}$ is a constant. The bulk glass-to-liquid transformation time exhibits the empirical relation (*103*):

$$\tau_{\text{trans}} \sim e^{\mathcal{A}_{\text{glass}}/(T-T_0)}. \tag{S16}$$

Despite their similar forms, Eq. S16 is for the melting transition time for glass, whereas VFT equation (Eq. S15) is for the bulk relaxation time for supercooled liquid.

Eq. S13 is insufficient to identify how l_{c} changes with the heating rate because v increases while τ_α decreases with the heating rate. Thus, we further derive the following equation to identify the influence of heating rate on l_{c} on the basis of Eqs. S13-S16 in the literature:

$$l_{\text{c}}(T) \sim e^{(\mathcal{A}_{\text{glass}}-\gamma\mathcal{A})/(T-T_0)}. \tag{S17}$$

$\mathcal{A}_{\text{glass}}$ increases with the glass stability and $\mathcal{A}_{\text{glass}} > \mathcal{A}$ (*103*), thus $\mathcal{A}_{\text{glass}} - \gamma\mathcal{A}$ increases with stability, resulting in a larger l_{c} for ultrastable glass than normal glass. Therefore surface melting is more difficult to observe for normal glass. Besides the stability effect, high heating rate causes high transition temperature T (*104*). Consequently, l_{c} is smaller under high heating rate, and surface melting is more difficult to observe.

F Figures

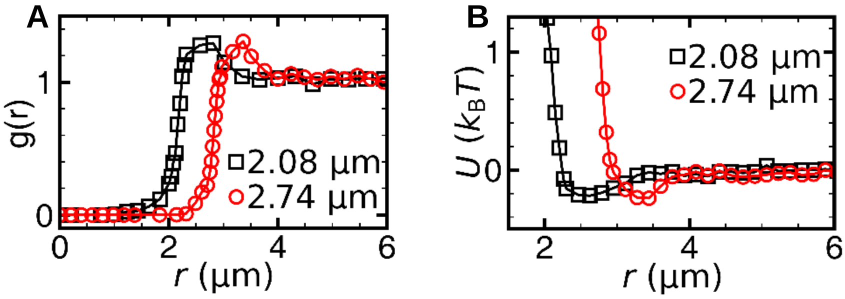

Fig. S 1. Radial distribution functions and corresponding pair potentials for dilute monolayer liquid at $T = 27.0$°C. (**A**), Radial distribution functions $g(r)$ for small spheres ($\sigma_a = 2.08$ µm) and large spheres ($\sigma_b = 2.74$ µm). They are used in the calculation of pair potentials. (**B**), Pair potential $U(r)$ for 2.08-µm-diameter and 2.74-µm-diameter PMMA spheres as function of center-to-center distance extracted from $g(r)$ in (**A**).

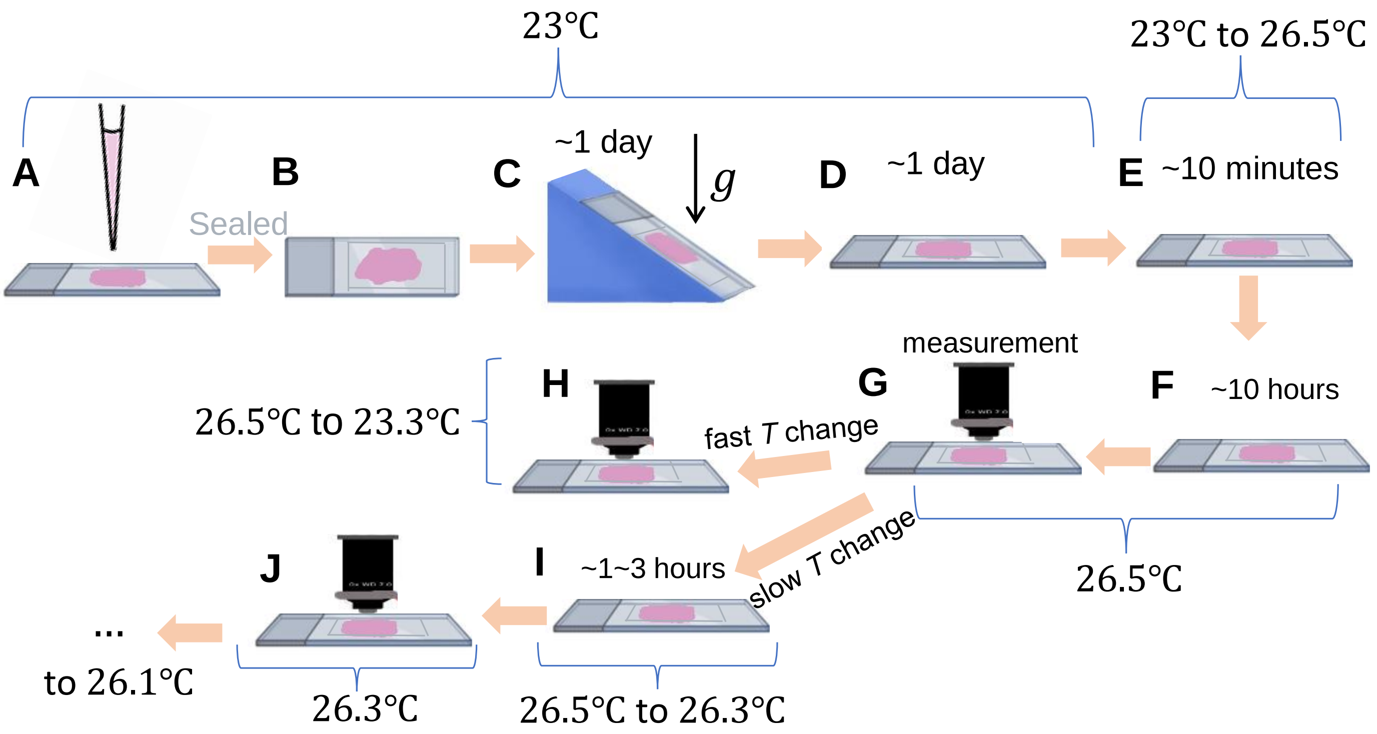

Fig. S 2. Schematic of the sample preparation.

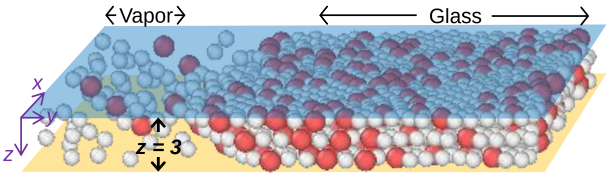

Fig. S 3. Schematic of a trilayer sample composed of large (red) and small (white) particles confined between two coverslips.

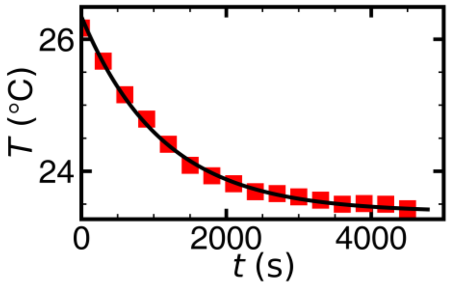

Fig. S 4. Temperature change of the monolayer glass in the main text after the temperature controller is set from 26.5°C to 23.3°C at $t = 0$ s. Solid curve: the exponential fit.

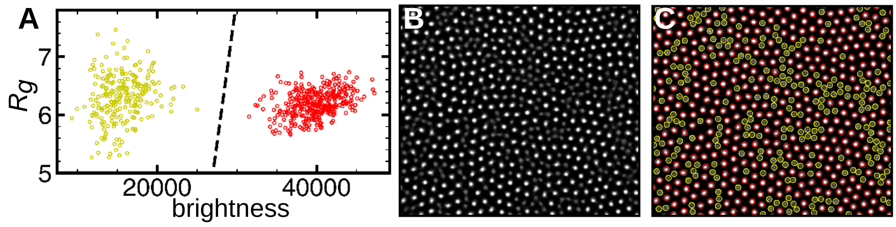

Fig. S 5. Identification of large and small spheres. (**A**), Large (red) and small (yellow) colloidal spheres have different radii of gyration and total brightness in the monolayer samples; thus, they can be well separated in the parameter space. (**B**), A raw image. (**C**), Criterion in (**A**) correctly identifies the large (red) and small (yellow) particles in (**B**).

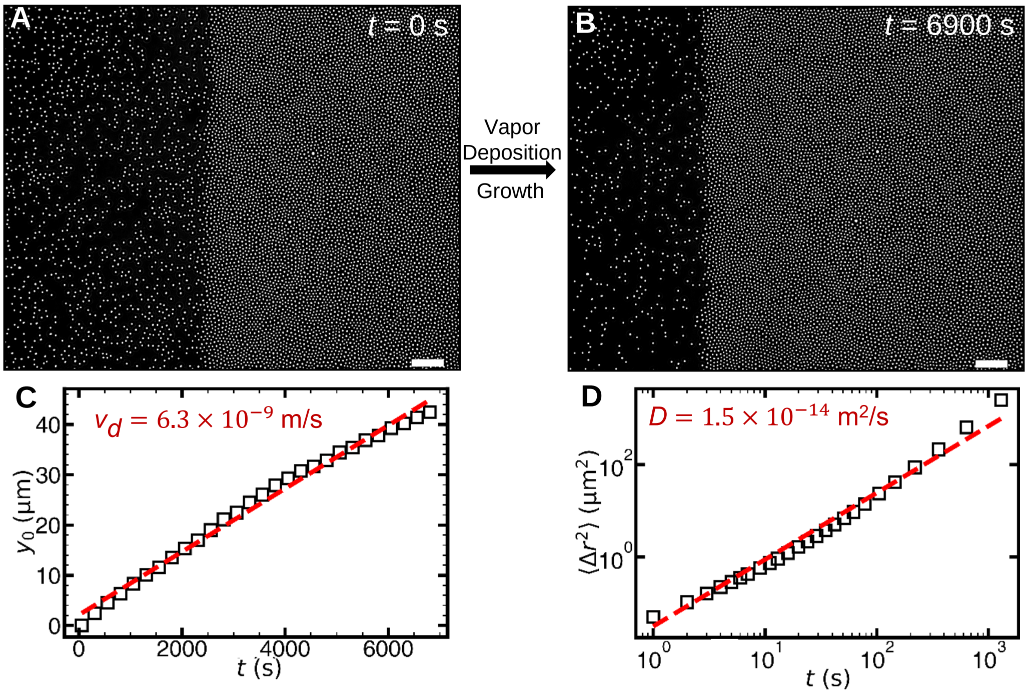

Fig. S 6. A monolayer glass grows via slow vapor deposition at 26.5°C. (**A**), $t = 0$ s. (**B**), $t = 6900$ s. Scale bars: 20 μm. (**C**), The position of the vapor–liquid interface y_0 during vapor deposition. The fitted slope gives the deposition rate $v_\mathrm{d} = 6.3 \times 10^{-9}$ m/s. (**D**), Mean-square displacement $\langle \Delta r^2 \rangle$ of particles at y_0 after the vapor deposition stage. The fitting gives the diffusion constant $D = \langle \Delta r^2 \rangle/(2t) = 1.5 \times 10^{-14}$ m^2/s.

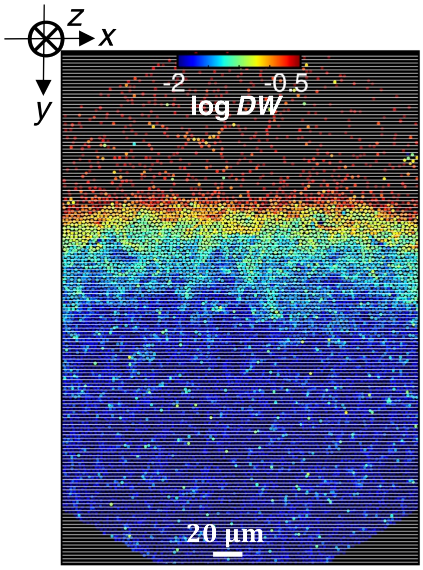

Fig. S 7. Monolayer sample at 27.0°C colored by $\log(DW)$ is divided into stripes parallel to the x direction. The dark corners are out of the field of view.

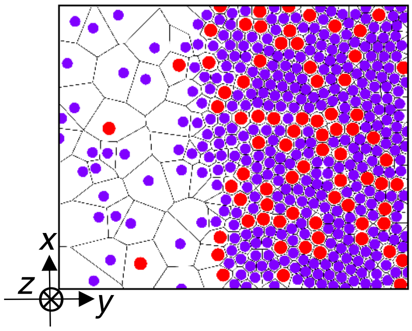

Fig. S 8. Example of the radical Voronoi tessellation in the monolayer of large (red) and small (purple) spheres.

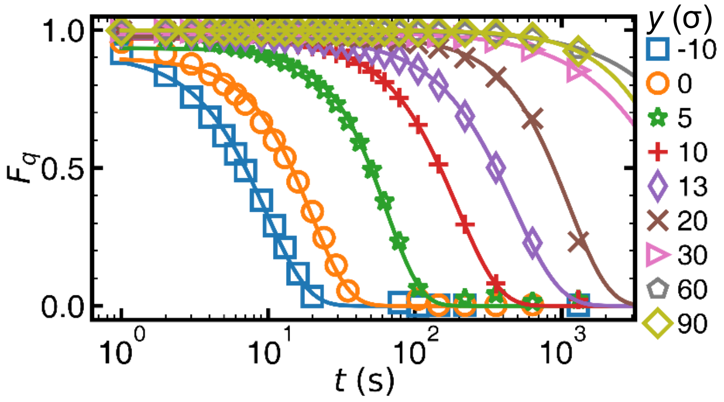

Fig. S 9. Self-intermediate scattering function at different depths y in the monolayer sample at 27.0°C. $F_q(t)$ for the deep bulk region has a much longer τ to decay to $1/e$ which is beyond our experimental time scale.

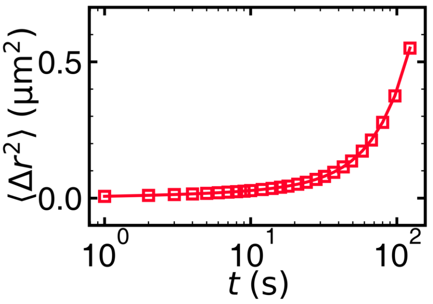

Fig. S 10. Mean square displacement at $y = 10\ \sigma$ and $T = 27.0$°C in the monolayer sample.

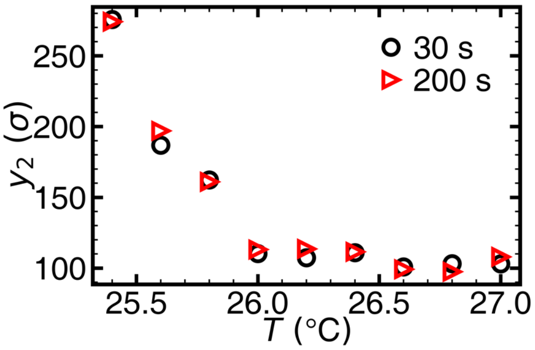

Fig. S 11. The interface between the glassy layer and bulk glass at y_2 determined by $\log(DW(y))$ with a time interval of 30 s (black circle) or 200 s (red triangle), respectively.

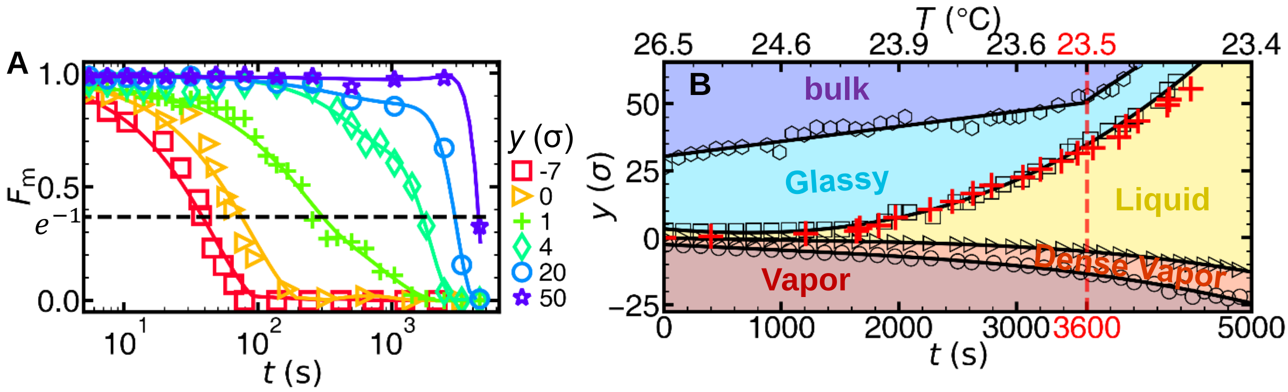

Fig. S 12. Melting interfaces defined by overlap function. (**A**), Overlap functions at different y give the melting interfaces at $F_\mathrm{m} = e^{-1}$. (**B**), Fig. 7H of the main text with the added melting interfaces (red crosses) measured from (**A**).

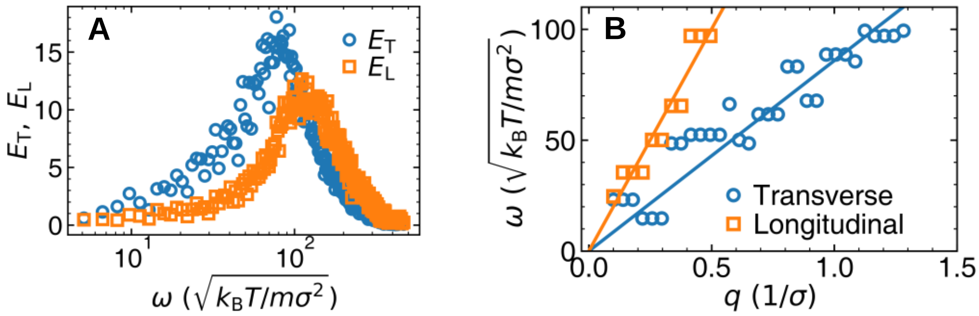

Fig. S 13. Dispersion relations for monolayer glass at $T = 26.6$°C. (**A**), Transverse and longitudinal spectral functions, $E_T(\omega)$ and $E_L(\omega)$, at $q = 0.89$. (**B**), Dispersion relations of transverse and longitudinal modes.

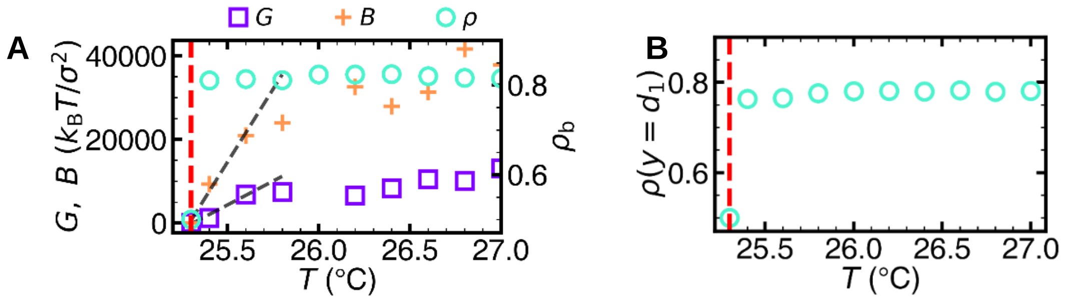

Fig. S 14. Bulk values and density at d_1 evolving with temperature. (**A**), For the monolayer glass under the slow temperature change, the bulk density ρ_{b} jumps at $T_{\mathrm{g}} = 25.3$°C (vertical dashed line) where the shear modulus G and bulk modulus B become zero. (**B**), Density at $y = d_1$ is nearly constant before melting (right hand side of the red dashed line).

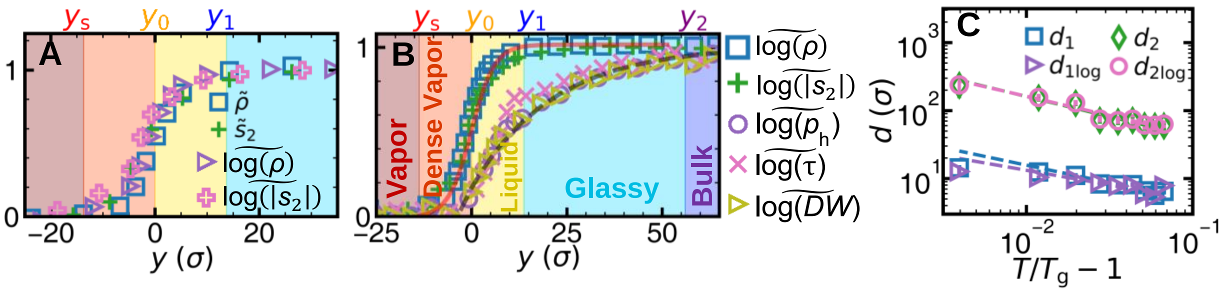

Fig. S 15. Comparisons between $\{\tilde{\rho}(y),\ \tilde{s}_2(y)\}$ and $\{\widetilde{\log(\rho(y))},\ \widetilde{\log(|s_2(y)|)}\}$. (**A**), Profiles of $\{\tilde{\rho}(y),\ \tilde{s}_2(y)\}$ versus $\{\widetilde{\log(\rho(y))},\ \widetilde{\log(|s_2(y)|)}\}$ for the monolayer at 26.0°C. (**B**), Profiles of $\widetilde{\log(\rho(y))}$, $\widetilde{\log(|s_2(y)|)}$, $\widetilde{\log(p_{\mathrm{h}}(y))}$, $\widetilde{\log(\tau(y))}$ and $\widetilde{\log(DW(y))}$ at 26.0°C, similar to Fig. 2E in the main text. (**C**), Layer thicknesses $d_{1,2\,\log}$ defined by $\widetilde{\log(\rho)}$ compared with $d_{1,2}$ defined by ρ.

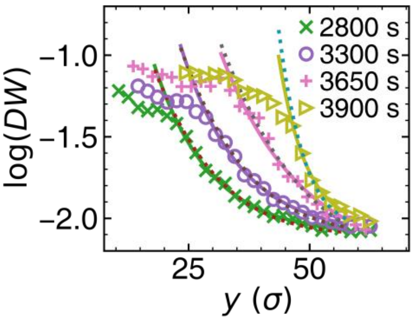

Fig. S 16. Profiles of $\log(DW)$ across the glassy layer at different times under the fast temperature change. Eq. 3 (solid curves) and Eq. 4 (dotted curves) of the main text fit well in the glassy layer and deviate in the liquid layer.

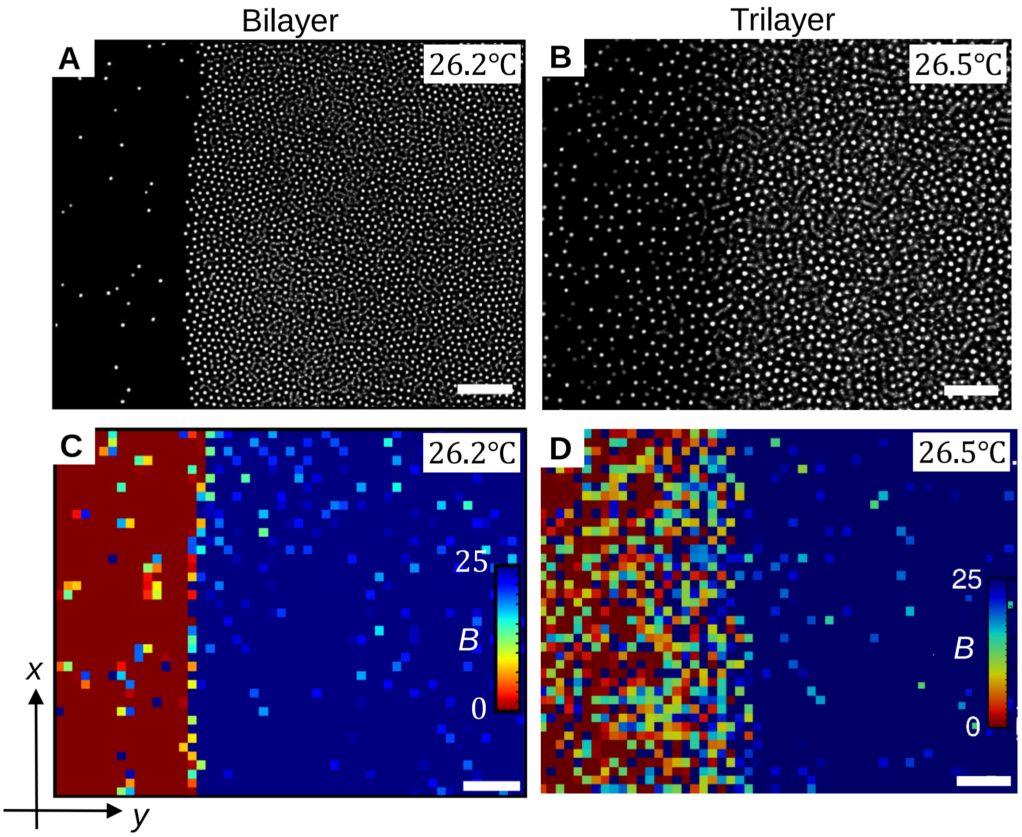

Fig. S 17. Coarse-grained pixel brightness and its fluctuation for multilayer samples. (**A, B**), Sample raw images of a bilayer sample and a trilayer sample, respectively. (**C, D**), Each coarse-grained 30×30 pixel^2 region in (**A, B**) is colored by its total brightness B. Scale bars: 20 μm.

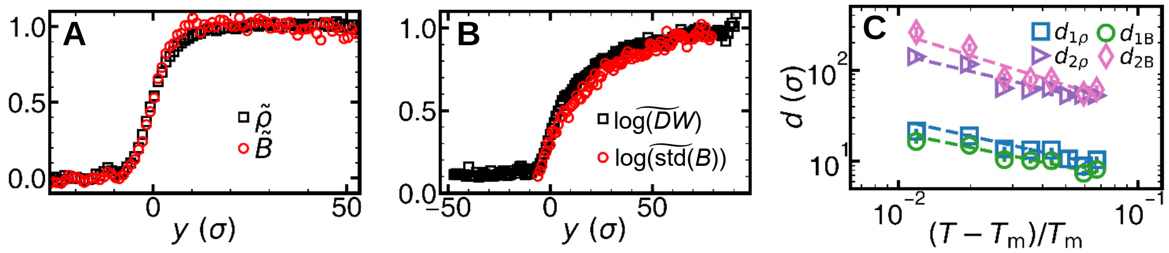

Fig. S 18. Reanalysis of the monolayer glass in main text Fig. 2 using the normalized coarse-grained pixel brightness $\tilde{B}$ and its normalized fluctuation $\log(\widetilde{\mathrm{std}}(B))$. (**A**), Structural parameters $\tilde{\rho}$ and $\tilde{B}$ exhibit the same profile. (**B**), Dynamic parameters $\log(\widetilde{DW})$ and $\log(\widetilde{\mathrm{std}}(B))$ exhibit the same profile in the liquid and glass regions. (**C**), The thicknesses $d_{1,2}$ of the surface liquid and glassy layers measured from the standard image analysis and from the coarse-grained pixel brightness, respectively. The two methods yield similar d which satisfies the same power law except for a slightly different prefactor.

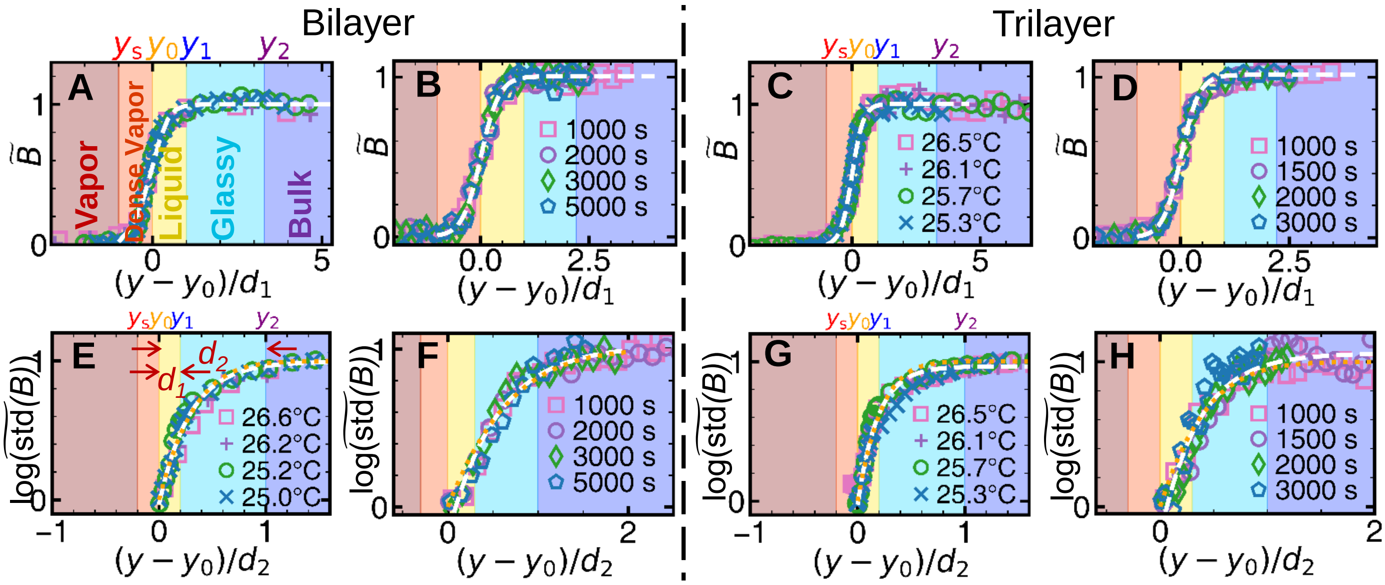

Fig. S 19. Collapsed profiles for the bilayer and trilayer under the slow and fast temperature change. Collapsed structural parameter $\tilde{B}(y)$ (**A–D**) and dynamic parameter $\log(\widetilde{\mathrm{std}(B)}(y))$ (**E–H**) for the bilayer (**A, B, E, F**) and trilayer (**C, D, G, H**) under the slow temperature change (**A, C, E, G**) and the fast temperature change (**B, D, F, H**). The white dashed curves are the fittings of Eq. 2 in the main text in (**A–D**) and the fittings of Eq. 3 in the main text in (**E–H**). The orange dotted curves are the fittings of Eq. 4 in the main text in (**E–H**). $d_{1,2}$ and $y_{s,0,1,2}$ are defined in Fig. 2E of the main text. (**A, E**) share the same legend. All panels share the same labels for the five colored regions. Similar plots for the monolayer sample are Figs. 4A, B, D, E and 6C, D of the main text.

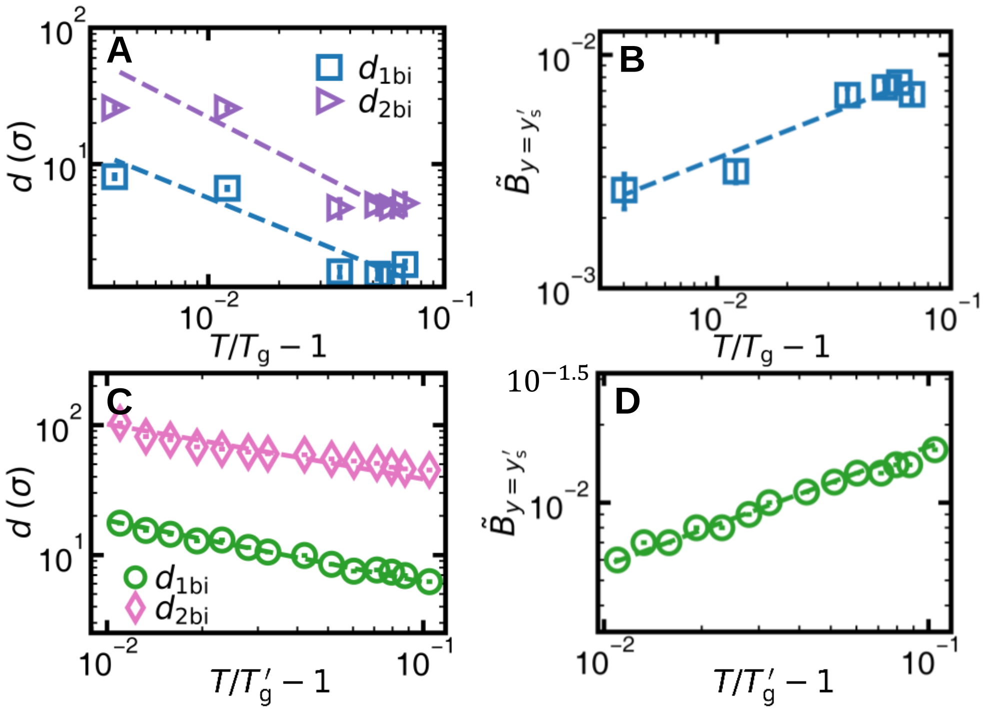

Fig. S 20. Power laws for the bilayer glass under the slow and fast temperature changes. Similar to Figs. 3 and 6A, B of the main text for the monolayer glass: (**A, C**), Layer thicknesses $d_{1,2}(T)$ fitted by $(T/T_{\rm g}-1)^{-\alpha_{1,2}}$ (main text Eq. 1, dashed lines) with $T_{\rm g}=24.9$℃, $\alpha_1=0.7\pm0.3$, $\alpha_2=0.9\pm0.2$ under the slow temperature change in (**A**), and $T'_{\rm g}=22.5$℃, $\alpha_1=0.47$, $\alpha_2=0.42$ under the fast temperature change in (**C**); (**B, D**), The effective density $\tilde{B}_{y=y'_{\rm s}}$ fitted by $(T/T_{\rm g}-1)^{\beta}$ (main text Eq. 6) with $\beta=0.39\pm0.11$ under the slow temperature change in (**B**) and $\beta=0.46$ under the fast temperature change in (**D**).

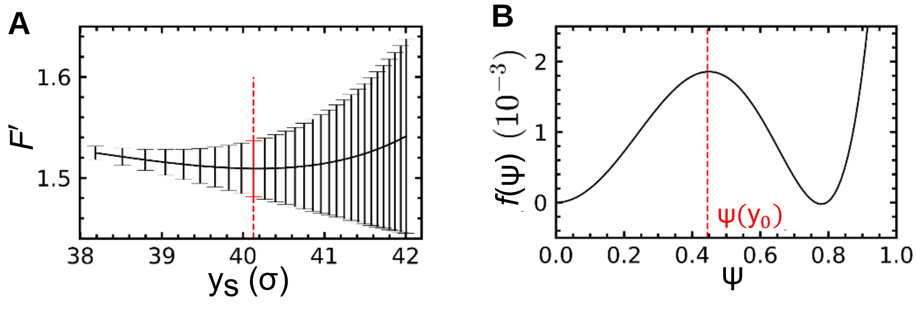

Fig. S 21. Definition of vapor and vapor-liquid interfaces by Landau theory. (**A**), System free energy F' in Eq. S9 under different choices of $y_{\rm s}$. Its minimum (red dashed line) gives the vapor interface $y_{\rm s}$ for the equilibrium state. (**B**), Bulk free energy in Eq. S5. Its maximum (red dashed line) gives the vapor–liquid interface y_0.

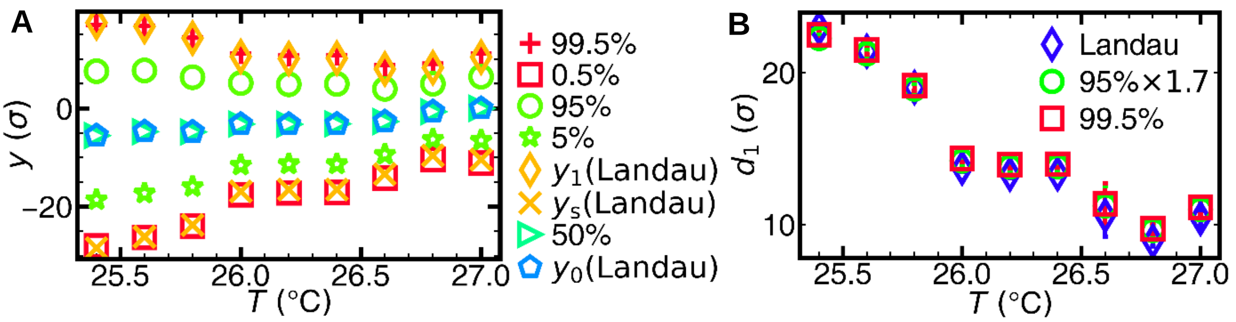

Fig. S 22. Comparing surface interfaces defined in different ways. (**A**), Interfaces' positions $y_{\mathrm{s},0,1}$ defined by $\tilde{\rho}(y = y_{\mathrm{s},0,1}) = 5\%, 50\%, 95\%$, by $\tilde{\rho}(y = y_{\mathrm{s},0,1}) = 0.5\%, 50\%, 99.5\%$ and by Landau theory in Fig. 21. The error bars are smaller than the symbols. (**B**), The surface liquid layer thicknesses defined by Landau theory and by $\tilde{\rho} = 99.5\%$ are the same but different from that defined by $\tilde{\rho} = 95\%$ by a constant prefactor of 1.7.

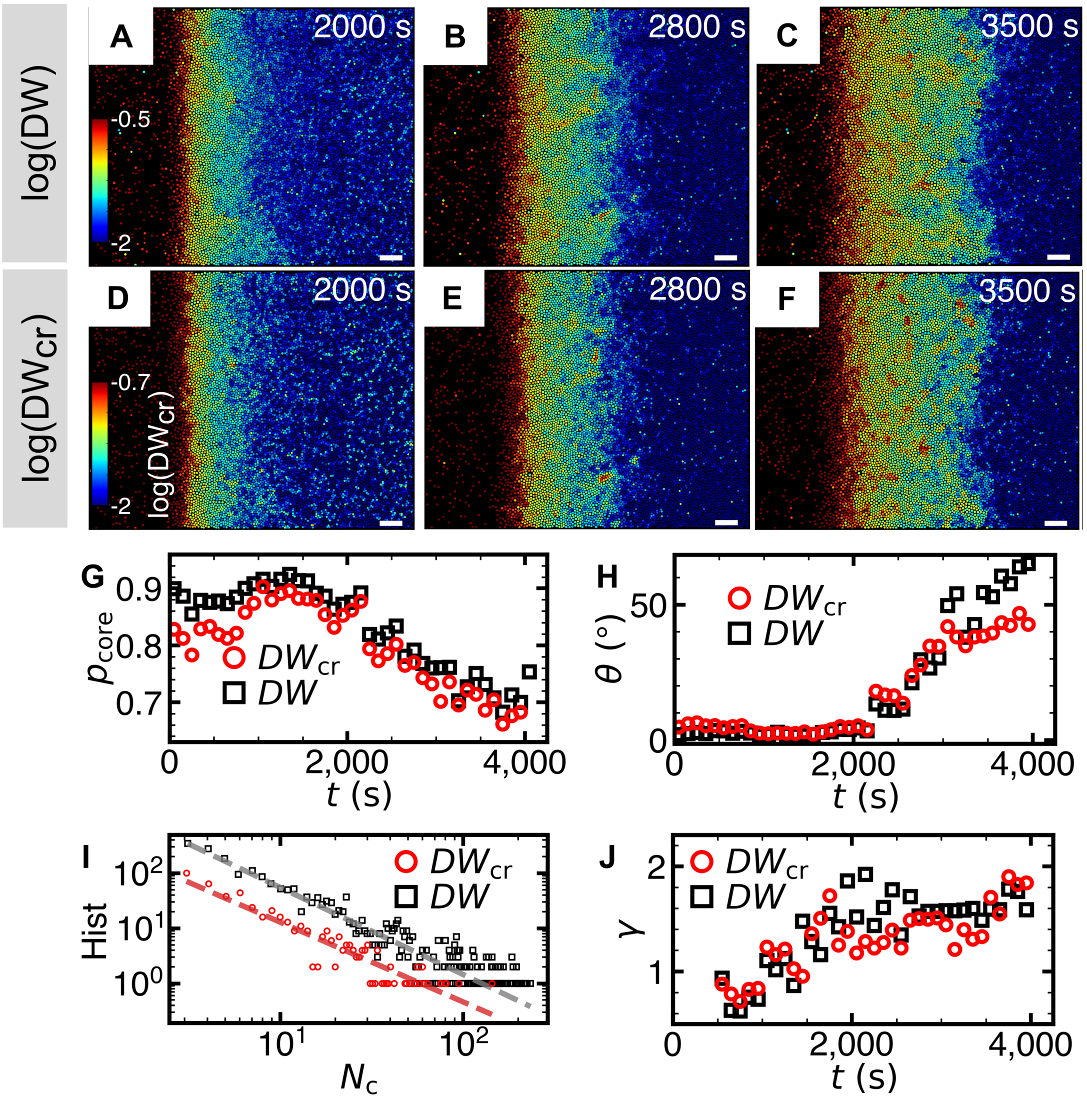

Fig. S 23. Comparison between results based on DW defined in Eq. 1 and the cage-relative DW_{cr} defined in Eq. 11 for monolayer glass under fast temperature change. (**A** to **F**), The surface regions colored by $\log(DW)$ in (**A** to **C**) and $\log(DW_{\mathrm{cr}})$ in (**D** to **F**) at 2000, 2800, and 3500 s, respectively. Scale bars: 20 µm. (**G** to **J**), Similar behavior for CRRs defined by the top 10% highest-DW in Figs. 9B, D, E, F and top 10% highest-DW_{cr} particles. (**G**), Evolution of the fraction of core-like particles in CRRs. (**H**), The orientation θ of CRR's long axis relative to the glass surface. θ is weighted average by CRR size and aspect ratio. (**I**), The histogram of CRR size N_{c} during [3000 s, 3100 s] fitted by the power law (dashed line) with the exponent $\gamma = 1.58$ for DW and $\gamma = 1.45$ for DW_{cr}. (**J**), $\gamma(t)$ from the size distributions of CRRs. Each data point is averaged over the time interval $[t - 50\text{ s}, t + 50\text{ s}]$.

G Movies

Movie S1. Typical vapor deposition growth at 26.5℃ before measurement.

Movie S2. Monolayer colloidal glass in Fig. 2 has completely melted at 25.3℃ under the slow temperature change. Large spheres are brighter. The slight expansion of the bulk (right-hand side) causes a drift in the melting.

Movie S3. The melting process of the monolayer colloidal glass under the fast temperature change, which corresponds to Fig. 7. Particles are colored by $\log(DW)$. The color bar is the same as that in Fig. 7A in the main text.

Movie S4. Evolution of $\log(\rho(y))$ and $-\log(DW(y))$ of the monolayer sample under the fast temperature change, which corresponds to Fig. 7D–F.

REFERENCES AND NOTES

1. M. Lulli, C.-S. Lee, H.-Y. Deng, C.-T. Yip, C.-H. Lam, Spatial heterogeneities in structural temperature cause Kovacs' expansion gap paradox in aging of glasses. *Phys. Rev. Lett.* **124**, 095501 (2020).

2. G. Biroli, J. P. Garrahan, Perspective: The glass transition. *J. Chem. Phys.* **138**, 12A301 (2013).

3. X. Monnier, D. Cangialosi, B. Ruta, R. Busch, I. Gallino, Vitrification decoupling from α-relaxation in a metallic glass. *Sci. Adv.* **6**, eaay1454 (2020).

4. M. Wang, K. Zhang, Z. Li, Y. Liu, J. Schroers, M. D. Shattuck, C. S. O'Hern, Asymmetric crystallization during cooling and heating in model glass-forming systems. *Phys. Rev. E Stat. Nonlin. Soft Matter Phys.* **91**, 032309 (2015).

5. S. F. Swallen, K. Traynor, R. J. McMahon, M. D. Ediger, T. E. Mates, Stable glass transformation to supercooled liquid via surface-initiated growth front. *Phys. Rev. Lett.* **102**, 065503 (2009).

6. C. Eisenmann, C. Kim, J. Mattsson, D. A. Weitz, Shear melting of a colloidal glass. *Phys. Rev. Lett.* **104**, 035502 (2010).

7. R. L. Jack, L. Berthier, The melting of stable glasses is governed by nucleation-and-growth dynamics. *J. Chem. Phys.* **144**, 244506 (2016).

8. E. Flenner, L. Berthier, P. Charbonneau, C. J. Fullerton, Front-mediated melting of isotropic ultrastable glasses. *Phys. Rev. Lett.* **123**, 175501 (2019).

9. A. Vila-Costa, J. Ràfols-Ribé, M. González-Silveira, A. F. Lopeandia, L. Abad-Muñoz, J. Rodríguez-Viejo, Nucleation and growth of the supercooled liquid phase control glass transition in bulk ultrastable glasses. *Phys. Rev. Lett.* **124**, 076002 (2020).

10. C. Rodríguez-Tinoco, M. Gonzalez-Silveira, J. Ràfols-Ribé, A. Vila-Costa, J. C. Martinez-Garcia, J. Rodríguez-Viejo, Surface-bulk interplay in vapor-deposited glasses: Crossover length and the origin of front transformation, *Phys. Rev. Lett.* **123**, 155501 (2019).

11. K. L. Kearns, M. D. Ediger, H. Huth, C. Schick, One micrometer length scale controls kinetic stability of low-energy glasses. *J. Phys. Chem. Lett.* **1**, 388–392 (2010).

12. A. Wisitsorasak, P. G. Wolynes, Fluctuating mobility generation and transport in glasses. *Phys. Rev. E Stat. Nonlin. Soft Matter Phys.* **88**, 022308 (2013).

13. R. Gutiérrez, J. P. Garrahan, Front propagation versus bulk relaxation in the annealing dynamics of a kinetically constrained model of ultrastable glasses. *J. Stat. Mech.* **2016**, 074005 (2016).

14. S. Léonard, P. Harrowell, Macroscopic facilitation of glassy relaxation kinetics: Ultrastable glass films with frontlike thermal response. *J. Chem. Phys.* **133**, 244502 (2010).

15. J. G. Dash, A. W. Rempel, J. S. Wettlaufer, The physics of premelted ice and its geophysical consequences. *Rev. Mod. Phys.* **78**, 695–741 (2006).

16. M. D. Ediger, Perspective: Highly stable vapor-deposited glasses. *J. Chem. Phys.* **147**, 210901 (2017).

17. Q. Zheng, Y. Zhang, M. Montazerian, O. Gulbiten, J. C. Mauro, E. D. Zanotto, Y. Yue, Understanding glass through differential scanning calorimetry. *Chem. Rev.* **119**, 7848–7939 (2019).

18. J. Ma, C. Yang, X. Liu, B. Shang, Q. He, F. Li, T. Wang, D. Wei, X. Liang, X. Wu, Y. Wang, F. Gong, P. Guan, W. Wang, Y. Yang, Fast surface dynamics enabled cold joining of metallic glasses. *Sci. Adv.* **5**, eaax7256 (2019).

19. R. D. Priestley, C. J. Ellison, L. J. Broadbelt, J. M. Torkelson, Structural relaxation of polymer glasses at surfaces, interfaces, and in between. *Science* **309**, 456–459 (2005).

20. M. D. Ediger, J. A. Forrest, Dynamics near free surfaces and the glass transition in thin polymer films: A view to the future. *Macromolecules* **47**, 471–478 (2014).

21. Y. Chai, T. Salez, J. D. McGraw, M. Benzaquen, K. Dalnoki-Veress, E. Raphael, J. A. Forrest, A direct quantitative measure of surface mobility in a glassy polymer. *Science* **343**, 994–999 (2014).

22. F. Chen, C.-H. Lam, O. K. C. Tsui, The surface mobility of glasses. *Science* **343**, 975–976 (2014).

23. B. Li, F. Wang, D. Zhou, Y. Peng, R. Ni, Y. Han, Modes of surface premelting in colloidal crystals composed of attractive particles. *Nature* **531**, 485–488 (2016).

24. G. L. Hunter, E. R. Weeks, The physics of the colloidal glass transition. *Rep. Prog. Phys.* **75**, 066501 (2012).

25. W. Kob, S. Roldán-Vargas, L. Berthier, Non-monotonic temperature evolution of dynamic correlations in glass-forming liquids. *Nat. Phys.* **8**, 164–167 (2012).

26. K. Hima Nagamanasa, S. Gokhale, A. K. Sood, R. Ganapathy, Direct measurements of growing amorphous order and non-monotonic dynamic correlations in a colloidal glass-former. *Nat. Phys.* **11**, 403–408 (2015).

27. X. Cao, H. Zhang, Y. Han, Release of free-volume bubbles by cooperative-rearrangement regions during the deposition growth of a colloidal glass. *Nat. Commun.* **8**, 362 (2017).

28. J. R. Savage, D. W. Blair, A. J. Levine, R. A. Guyer, A. D. Dinsmore, Imaging the sublimation dynamics of colloidal crystallites. *Science* **314**, 795–798 (2006).

29. D. Bonn, J. Otwinowski, S. Sacanna, H. Guo, G. Wegdam, P. Schall, Direct observation of colloidal aggregation by critical Casimir forces. *Phys. Rev. Lett.* **103**, 156101 (2009).

30. P. J. Santos, P. A. Gabrys, L. Z. Zornberg, M. S. Lee, R. J. Macfarlane, Macroscopic materials assembled from nanoparticle superlattices. *Nature* **591**, 586–591 (2021).

31. A. Yethiraj, A. van Blaaderen, A colloidal model system with an interaction tunable from hard sphere to soft and dipolar. *Nature* **421**, 513–517 (2003).

32. S. F. Swallen, K. L. Kearns, M. K. Mapes, Y. S. Kim, R. J. McMahon, M. D. Ediger, T. Wu, L. Yu, S. Satija, Organic glasses with exceptional thermodynamic and kinetic stability. *Science* **315**, 353–356 (2007).

33. J. C. Crocker, D. G. Grier, Methods of digital video microscopy for colloidal studies. *J. Colloid Interface Sci.* **179**, 298–310 (1996).

34. A. Baranyai, D. J. Evans, Direct entropy calculation from computer simulation of liquids. *Phys. Rev. A* **40**, 3817–3822 (1989).

35. D. M. Sussman, S. S. Schoenholz, E. D. Cubuk, A. J. Liu, Disconnecting structure and dynamics in glassy thin films. *Proc. Natl. Acad. Sci. U.S.A.* **114**, 10601–10605 (2017).

36. H. Tanaka, T. Kawasaki, H. Shintani, K. Watanabe, Critical-like behaviour of glass-forming liquids. *Nat. Mater.* **9**, 324–331 (2010).

37. R. E. Nettleton, M. S. Green, Expression in terms of molecular distribution functions for the entropy density in an infinite system. *J. Chem. Phys.* **29**, 1365–1370 (1958).

38. I. Borzsák, A. Baranyai, On the convergence of Green's entropy expansion. *Chem. Rev.* **165**, 227–230 (1992).

39. S. Mazoyer, F. Ebert, G. Maret, P. Keim, Dynamics of particles and cages in an experimental 2D glass former. *Europhys. Lett.* **88**, 66004 (2009).

40. S. Vivek, C. P. Kelleher, P. M. Chaikin, E. R. Weeks, Long-wavelength fluctuations and the glass transition in two dimensions and three dimensions. *Proc. Natl. Acad. Sci. U.S.A.* **114**, 1850–1855 (2017).

41. H. Shiba, Y. Yamada, T. Kawasaki, K. Kim, Unveiling dimensionality dependence of glassy dynamics: 2D infinite fluctuation eclipses inherent structural relaxation. *Phys. Rev. Lett.* **117**, 245701 (2016).

42. S. S. Schoenholz, E. D. Cubuk, D. M. Sussman, E. Kaxiras, A. J. Liu, A structural approach to relaxation in glassy liquids. *Nat. Phys.* **12**, 469–471 (2016).

43. J. Mattsson, H. M. Wyss, A. Fernandez-Nieves, K. Miyazaki, Z. Hu, D. R. Reichman, D. A. Weitz, Soft colloids make strong glasses. *Nature* **462**, 83–86 (2009).

44. H. Yuan, J. Yan, P. Gao, S. K. Kumar, O. K. C. Tsui, Microscale mobile surface double layer in a glassy polymer. *Sci. Adv.* **8**, eabq5295 (2022).

45. R. Lipowsky, W. Speth, Semi-infinite systems with first-order bulk transitions. *Phys. Rev. B* **28**, 3983–3993 (1983).

46. X. Wang, B. Li, X. Xu, Y. Han, Surface roughening, premelting and melting of monolayer and bilayer crystals. *Soft Matter* **17**, 688–693 (2021).

47. G. Hantal, B. Fábián, M. Sega, P. Jedlovszky, Contribution of the two liquid phases to the interfacial tension at various water-organic liquid-liquid interfaces. *J. Mol. Liq.* **306**, 112872 (2020).

48. Y. Yang, M. Asta, B. B. Laird, Solid-liquid interfacial premelting. *Phys. Rev. Lett.* **110**, 096102 (2013).

49. N. P. Kryuchkov, N. A. Dmitryuk, W. Li, P. V. Ovcharov, Y. Han, A. V. Sapelkin, S. O. Yurchenko, Mean-field model of melting in superheated crystals based on a single experimentally measurable order parameter. *Sci. Rep.* **11**, 17963 (2021).

50. W. Schweika, H. Reichert, W. Babik, O. Klein, S. Engemann, Strain-induced incomplete wetting at CuAu(001) surfaces, *Phys. Rev. B* **70**, 041401 (2004).

51. J. E. Mueller, J. W. Gillespie Jr., S. G. Advani, Effects of interaction volume on x-ray line-scans across an ultrasonically consolidated aluminum/copper interface. *Scanning* **35**, 327–335 (2013).

52. Z. Fakhraai, J. A. Forrest, Measuring the surface dynamics of glassy polymers. *Science* **319**, 600–604 (2008).

53. K. S. Schweizer, D. S. Simmons, Progress towards a phenomenological picture and theoretical understanding of glassy dynamics and vitrification near interfaces and under nanoconfinement. *J. Chem. Phys.* **151**, 240901 (2019).

54. N. B. Tito, J. E. G. Lipson, S. T. Milner, Lattice model of mobility at interfaces: Free surfaces, substrates, and bilayers. *Soft Matter* **9**, 9403 (2013).

55. T. Salez, J. Salez, K. Dalnoki-Veress, E. Raphaël, J. A. Forrest, Cooperative strings and glassy interfaces. *Proc. Natl. Acad. Sci. U.S.A.* **112**, 8227–8231 (2015).

56. L. Berthier, G. Biroli, Theoretical perspective on the glass transition and amorphous materials. *Rev. Mod. Phys.* **83**, 587–645 (2011).

57. L. Santen, W. Krauth, Absence of thermodynamic phase transition in a model glass former. *Nature* **405**, 550–551 (2000).

58. F. Weysser, D. Hajnal, Tests of mode-coupling theory in two dimensions. *Phys. Rev. E Stat. Nonlin. Soft Matter Phys.* **83**, 041503 (2011).

59. Z. Zheng, R. Ni, F. Wang, M. Dijkstra, Y. Wang, Y. Han, Structural signatures of dynamic heterogeneities in monolayers of colloidal ellipsoids. *Nat. Commun.* **5**, 3829 (2014).

60. P. Gallo, M. Rovere, Relation between the two-body entropy and the relaxation time in supercooled water. *Phys. Rev. E Stat. Nonlin. Soft Matter Phys.* **91**, 012107 (2015).

61. C. Zhang, L. Hu, Y. Yue J. C. Mauro, Fragile-to-strong transition in metallic glass-forming liquids. *J. Chem. Phys.* **133**, 014508 (2010).

62. F. Mallamace, C. Branca, C. Corsaro, N. Leone, J. Spooren, S.-H. Chen, H. E. Stanley, Transport properties of glass-forming liquids suggest that dynamic crossover temperature is as important as the glass transition temperature. *Proc. Natl. Acad. Sci. U.S.A.* **107**, 22457–22462 (2010).

63. H. Tanaka, H. Tong, R. Shi, J. Russo, Revealing key structural features hidden in liquids and glasses. *Nat. Rev. Phys.* **1**, 333–348 (2019).

64. S. Alvarado, M. Campagna, H. Hopster, Surface magnetism of Ni(100) near the critical region by spin-polarized electron scattering. *Phys. Rev. Lett.* **48**, 51–54 (1982).

65. H. Löwen, Melting, freezing and colloidal suspensions. *Phys. Rep.* **237**, 249–324 (1994).

66. J. D. Stevenson, J. Schmalian, P. G. Wolynes, The shapes of cooperatively rearranging regions in glass-forming liquids. *Nat. Phys.* **2**, 268–274 (2006).

67. Z. Zheng, R. Ni, Y. Wang, Y. Han, Translational and rotational critical-like behaviors in the glass transition of colloidal ellipsoid monolayers. *Sci. Adv.* **7**, eabd1958 (2021).

68. H. Peng, H. Liu, T. Voigtmann, Non-monotonic dynamic correlations beneath the surface of glass-forming liquids. *Phys. Rev. Lett.* **129**, 215501 (2021).

69. T. Lay, T. C. Wallace, Modern Global Seismology, in *International Geophysics Series* (Academic Press, San Diego, 1995), vol. 58.

70. D. R. Reid, I. Lyubimov, M. D. Ediger, J. J. de Pablo, Age and structure of a model vapour-deposited glass. *Nat. Commun.* **7**, 13062 (2016).

71. C. Rodríguez-Tinoco, M. González-Silveira, M. Barrio, P. Lloveras, J. L. Tamarit, J.-L. Garden, J. Rodríguez-Viejo, Ultrastable glasses portray similar behaviour to ordinary glasses at high pressure. *Sci. Rep.* **6**, 34296 (2016).

72. J. A. Forrest, K. Dalnoki-Veress, The glass transition in thin polymer films. *Adv. Colloid Interface Sci.* **94**, 167–195 (2001).

73. Z. Hao, A. Ghanekarade, N. Zhu, K. Randazzo, D. Kawaguchi, K. Tanaka, X. Wang, D. S. Simmons, R. D. Priestley, B. Zuo, Mobility gradients yield rubbery surfaces on top of polymer glasses. *Nature* **596**, 372–376 (2021).

74. S. H. Behrens, D. G. Grier, Pair interaction of charged colloidal spheres near a charged wall. *Phys. Rev. E Stat. Nonlin. Soft Matter Phys.* **64**, 050401 (2001).

75. M. Polin, D. G. Grier, Y. Han, Colloidal electrostatic interactions near a conducting surface. *Phys. Rev. E Stat. Nonlin. Soft Matter Phys.* **76**, 041406 (2007).

76. E. M. Chan, Two-dimensional Born-Green-Yvon and other integral equations. *J. Phys. C: Solid State Phys.* **10**, 3477–3486 (1977).

77. R. Ganapathy, M. R. Buckley, S. J. Gerbode, I. Cohen, Direct measurements of island growth and step-edge barriers in colloidal epitaxy. *Science* **327**, 445–448 (2010).

78. W. van Megen, T. C. Mortensen, S. R. Williams, J. Müller, Measurement of the self-intermediate scattering function of suspensions of hard spherical particles near the glass transition. *Phys. Rev. E Stat. Nonlin. Soft Matter Phys.* **58**, 6073–6085 (1998).

79. A. Samanta, S. M. Ali, S. K. Ghosh, New universal scaling laws of diffusion and Kolmogorov-Sinai entropy in simple liquids. *Phys. Rev. Lett.* **92**, 145901 (2004).

80. P. Luo, C. R. Cao, F. Zhu, Y. M. Lv, Y. H. Liu, P. Wen, H. Y. Bai, G. Vaughan, M. di Michiel, B. Ruta, W. H. Wang, Ultrastable metallic glasses formed on cold substrates. *Nat. Commun.* **9**, 1389 (2018).

81. H. Yoon, G. B. McKenna, Testing the paradigm of an ideal glass transition: Dynamics of an ultrastable polymeric glass. *Sci. Adv.* **4**, eaau5423 (2018).

82. L. Berthier, P. Charbonneau, E. Flenner, F. Zamponi, Origin of ultrastability in vapor-deposited glasses. *Phys. Rev. Lett.* **119**, 188002 (2017).

83. K. L. Ngai, M. Paluch, C. Rodríguez-Tinoco, Why is surface diffusion the same in ultrastable, ordinary, aged, and ultrathin molecular glasses? *Phys. Chem. Chem. Phys.* **19**, 29905–29912 (2017).

84. W. W. Mullins, Flattening of a nearly plane solid surface due to capillarity. *J. Appl. Phys.* **30**, 77–83 (1959).

85. Y. Li, W. Zhang, C. Bishop, C. Huang, M. D. Ediger, L. Yu, Surface diffusion in glasses of rod-like molecules posaconazole and itraconazole: Effect of interfacial molecular alignment and bulk penetration. *Soft Matter* **16**, 5062–5070 (2020).

86. Y. Zhang, Z. Fakhraai, Invariant fast diffusion on the surfaces of ultrastable and aged molecular glasses. *Phys. Rev. Lett.* **118**, 066101 (2017).

87. K. L. Kearns, P. Krzyskowski, Z. Devereaux, Using deposition rate to increase the thermal and kinetic stability of vapor-deposited hole transport layer glasses via a simple sublimation apparatus. *J. Chem. Phys.* **146**, 203328 (2017).

88. R. Blumenfeld, Disorder criterion and explicit solution for the disc random packing problem. *Phys. Rev. Lett.* **127**, 118002 (2021).

89. C. L. Klix, G. Maret, P. Keim, Discontinuous shear modulus determines the glass transition temperature. *Phys. Rev. X* **5**, 041033 (2015).

90. C. L. Klix, F. Ebert, F. Weysser, M. Fuchs, G. Maret, P. Keim, Glass elasticity from particle trajectories. *Phys. Rev. Lett.* **109**, 178301 (2012).

91. C. Rycroft, "Voro++: A three-dimensional voronoi cell library in C++" [Technical Report, Lawrence Berkeley National Lab. (LBNL), 2009].

92. L. Berthier, M. D. Ediger, How to "measure" a structural relaxation time that is too long to be measured? *J. Chem. Phys.* **153**, 044501 (2020).

93. H. Shintani, H. Tanaka, Universal link between the boson peak and transverse phonons in glass. *Nat. Mater.* **7**, 870–877 (2008).

94. S. Toxvaerd, J. Stecki, Density profiles at a planar liquid-liquid interface. *J. Chem. Phys.* **102**, 7163–7168 (1995).

95. S. Toxværd, *Statistical Mechanics*, K. Singer, Ed. (Royal Society of Chemistry, Cambridge, 1975), vol. 2, pp. 256–299.

96. H. Ramalingam, M. Asta, A. van de Walle, J. J. Hoyt, Atomic-scale simulation study of equilibrium solute adsorption at alloy solid-liquid interfaces, *Interface Sci.* **10**, 149–158 (2002).

97. D. Danilov, B. Nestler, M. Guerdane, H. Teichler, Bridging the gap between molecular dynamics simulations and phase-field modelling: Dynamics of a $[Ni_xZr_{1-x}]_{liquid}$–$Zr_{crystal}$ solidification front. *J. Phys. D: Appl. Phys.* **42**, 015310 (2009).

98. E. S. Wu, W. W. Webb, Critical liquid-vapor interface in SF_6. I. Thickness of the diffuse transition layer. *Phys. Rev. A* **8**, 2065–2076 (1973).

99. S. Fisk, B. Widom, Structure and free energy of the interface between fluid phases in equilibrium near the critical point. *J. Chem. Phys.* **50**, 3219–3227 (1969).

100. J. G. Dash, Surface melting. *Contemp. Phys.* **30**, 89–100 (1989).

101. A. Sepúlveda, S. F. Swallen, L. A. Kopff, R. J. McMahon, M. D. Ediger, Stable glasses of indomethacin and α,α,β-tris-naphthylbenzene transform into ordinary supercooled liquids. *J. Chem. Phys.* **137**, 204508 (2012).

102. C. Rodríguez-Tinoco, M. Gonzalez-Silveira, J. Ràfols-Ribé, A. F. Lopeandía, M. T. Clavaguera-Mora, J. Rodríguez-Viejo, Evaluation of growth front velocity in ultrastable glasses of Indomethacin over a wide temperature interval. *J. Phys. Chem. B* **118**, 10795–10801 (2014).

103. C. Rodríguez-Tinoco, J. Ràfols-Ribé, M. González-Silveira, J. Rodríguez-Viejo, Relaxation dynamics of glasses along a wide stability and temperature range. *Sci. Rep.* **6**, 35607 (2016).

104. C. T. Moynihan, A. J. Easteal, J. Wilder, J. Tucker, Dependence of the glass transition temperature on heating and cooling rate. *J. Phys. Chem.* **78**, 2673–2677 (1974).
